# THSD4 promotes hair growth by facilitating dermal papilla and hair matrix interactions

**DOI:** 10.7150/thno.103221

**Published:** 2025-02-25

**Authors:** Miaomiao Wang, Mengyue Wang, Jingwei Jiang, Ke Li, Huan Liang, Nian'ou Wang, Yi Zou, Dehuan Wang, Siyi Zhou, Yuchun Tang, Wang Wu, Weiming Qiu, Xinxin Li, Xusheng Wang, Qiaoli Xie, Xiao Xiang, Wei Zhou, Li Yang, Cheng-Ming Chuong, Mingxing Lei

**Affiliations:** 1 Key Laboratory of Biorheological Science and Technology of Ministry of Education &111 Project Laboratory of Biomechanics and Tissue Repair, College of Bioengineering, Chongqing University, Chongqing 400044, China.; 2 Radiation Oncology Center, Chongqing University Cancer Hospital, Chongqing, China.; 3 Shenzhen Accompany Technology Cooperation, ltd, Shenzhen 518000, China.; 4 Department of Burns and Plastic Surgery, Wuhan General Hospital of Chinese People's Liberation Army, Wuhan 430000, China.; 5 School of Pharmaceutical Sciences (Shenzhen), Shenzhen Campus of Sun Yat-Sen University, Sun Yat-Sen University, Shenzhen 518107, China.; 6 Department of Pathology, Keck School of Medicine, University of Southern California, Los Angeles, CA 90033, USA.

**Keywords:** aging, dermal papilla, extracellular matrix, epithelial-mesenchymal interaction, low-temperature treatment

## Abstract

**Introduction:** Aging causes striking changes in the extracellular matrix (ECM) in hair follicles, which has a profound influence on hair growth. How the ECM of dermal papilla (DP), the master regulator of hair growth, changes during aging remains largely unknown.

**Methods:** Herovici staining, Western Blotting and immunofluorescence were used to assess DP ECM and protein expression in hair follicles. Bulk and single cell RNA-sequencing were used to analyze gene expression and predict upstream and downstream regulators of target genes. Skin organoid and mouse models were used for functional validation of molecular mechanisms.

**Results:** Aged follicle DP shows drastic depletion of ECM in which Thrombospondin Type 1 Domain Containing 4 (Thsd4) is highly downregulated. THSD4 is specifically expressed in the interface between DP and hair matrix (HM). It promotes hair growth by enhancing the interaction between dermal (DP) and epithelial cells (HM) through the SDC4-THSD4-CXCL1 signaling axis in both skin organoids and mouse models. Murine dorsal hair follicles show upregulated THSD4, enhanced DP-HM interaction, and hair growth following exposure to low temperature.

**Conclusions:** THSD4 is a key micro- and macro-environmental mediator to promote hair growth by facilitating epidermal-mesenchymal interactions during aging. These findings demonstrate the therapeutic potential of low-temperature treatment for treating unwanted hair loss.

## Introduction

Aging is characterized by a gradual loss of tissue integrity, leading to impaired biological function, increased susceptibility to diseases, and eventual death 1. Over time, most organs in mammals tend to shrink or thin out, which is associated with a decline in functionality and regenerative capacity 2. The effects of aging are particularly prominent in the skin, with loss of skin appendages such as hair follicles and the onset of wrinkles, laxity, and atrophy 3. The hair follicle serves as an excellent model to investigate aging, as hair thinning, loss, and hair graying, as well as follicular atrophy, are common manifestations of aging of cells in the hair follicles 4-6. These are largely implicated in the alterations of hair follicle stem cells (HFSCs) and particularly their mesenchymal niche called dermal papilla (DP) 7, 8.

As the major regulatory center, the DP is influenced by a plethora of molecular signals during the development, growth, cycle, and aging of hair follicles 9-13. We have previously proposed that hair follicle aging, reflected by the self-renewal and activation capacities of HFSCs, is determined by the accumulation of both intrinsic and extrinsic factors 14. Intrinsic factors such as the epigenetic landscape 15, determinants of stemness 15, 16, and the careful balance of biochemical signals 17, 18 all contribute to the alteration of hair cycle and growth during aging. Nonetheless, extrinsic factors such as the extracellular matrix (ECM), a crucial component of the HFSC niche, can also have a profound influence on hair follicle homeostasis and hair growth 19. The controlled release of ECM molecules can effectively activate HFSCs both *in vivo* and *in vitro*, resulting in enhanced hair follicle regeneration 20. However, the depletion of ECM components, such as the hydrolysis of Type XVII collagen due to accumulation of DNA damage, can re-direct HFSC differentiation into the epidermal lineage, leading to hair follicle atrophy and loss during aging 21. ECM also serves as a mechanical stimulus that influences the stiffness of the HFSC microenvironment. For instance, increased basement membrane stiffness results in transcriptional repression of genes important for hair regeneration during aging 22, whereas a decrease in the stiffness of hair germs, such as reduction in their actomyosin contractibility by MicroRNA-205, is required for cell cycle re-entry and subsequent hair regeneration 23.

The prerequisite for hair follicle development is the formation of dermal condensates where fibronectin enhances cell adhesion and maintains high cell motility to promote DP cell aggregation 24. Once established, the DP becomes enriched in basement membrane components. As one such component, Lama5 (Laminin subunit alpha 5) between the hair germ and DP is critical for hair regeneration. Hair follicles of the Lama5 knockout mice have been shown to have reduced linkage between the hair germ and DP junction, which significantly thwarted hair regeneration 25. Moreover, the ECM in the mesenchymal niche may also regulate the terminal or vellus hair formation, as the thicker terminal follicles are richer in ECM compared to that of the thinner vellus follicles 26. Nevertheless, despite unprecedented advancements in hair follicle aging research in recent years 1, how alterations of DP ECM during aging influence hair follicle growth remains elusive.

In addition to the extrinsic factors of the follicular micro-environment, alternations of the external factors in the outside environment can also contribute to the regulation of hair growth and cycle 21, 27-30. For instance, human hair shedding and cycle exhibit a clear pattern of seasonality. More hairs are shed in the summer when the ambient temperature is high compared to spring and winter when the ambient temperatures are relatively lower. Coincidentally, there is a greater number of hair follicles in the anagen (hair growth phase) compared to that in the spring and winter 31. Thus, can temperature modulate hair growth during skin aging?

In this study, we investigated the impact of aging on DP ECM and discovered epidermal-mesenchymal interaction as a major contributor to the age- and external environment-induced change in follicular function. We characterized the overall ECM changes in young and aged mouse vibrissae and dorsal skin hair as well as human scalp hair follicles. Analyses of bulk- and single-cell RNA-sequencing data (RNA-seq and scRNA-seq, respectively) and Western blotting (WB) revealed a novel ECM component, THSD4, which became significantly downregulated in the DP upon aging. THSD4 was expressed in the boundary of DP and hair matrix (HM) and promoted hair growth from aging follicles by enhancing DP-HM interaction via Syndecan 4 (SDC4) and chemokine (C-X-C motif) ligand 1 (CXCL1). In addition to micro-environmental changes, we found that transient (5 min) exposure to low temperature (5 ºC) triggered Type I and III neocollagenesis and THSD4 expression which led to increased hair bulb size and DP area, both are indicative of a proliferative hair follicle. These findings advance our understanding of how DP ECM influences hair follicle growth and offer potential avenues for addressing issues such as hair loss during aging by applying low-temperature treatment. Slowing the aging process of hair follicles by modulation of ECM components could potentially provide clinical guidance for sustained follicle growth and prevention of unwanted hair loss.

## Results

### Decreased THSD4 expression in the DP-HM junction during hair follicle aging

**Figure 1:**
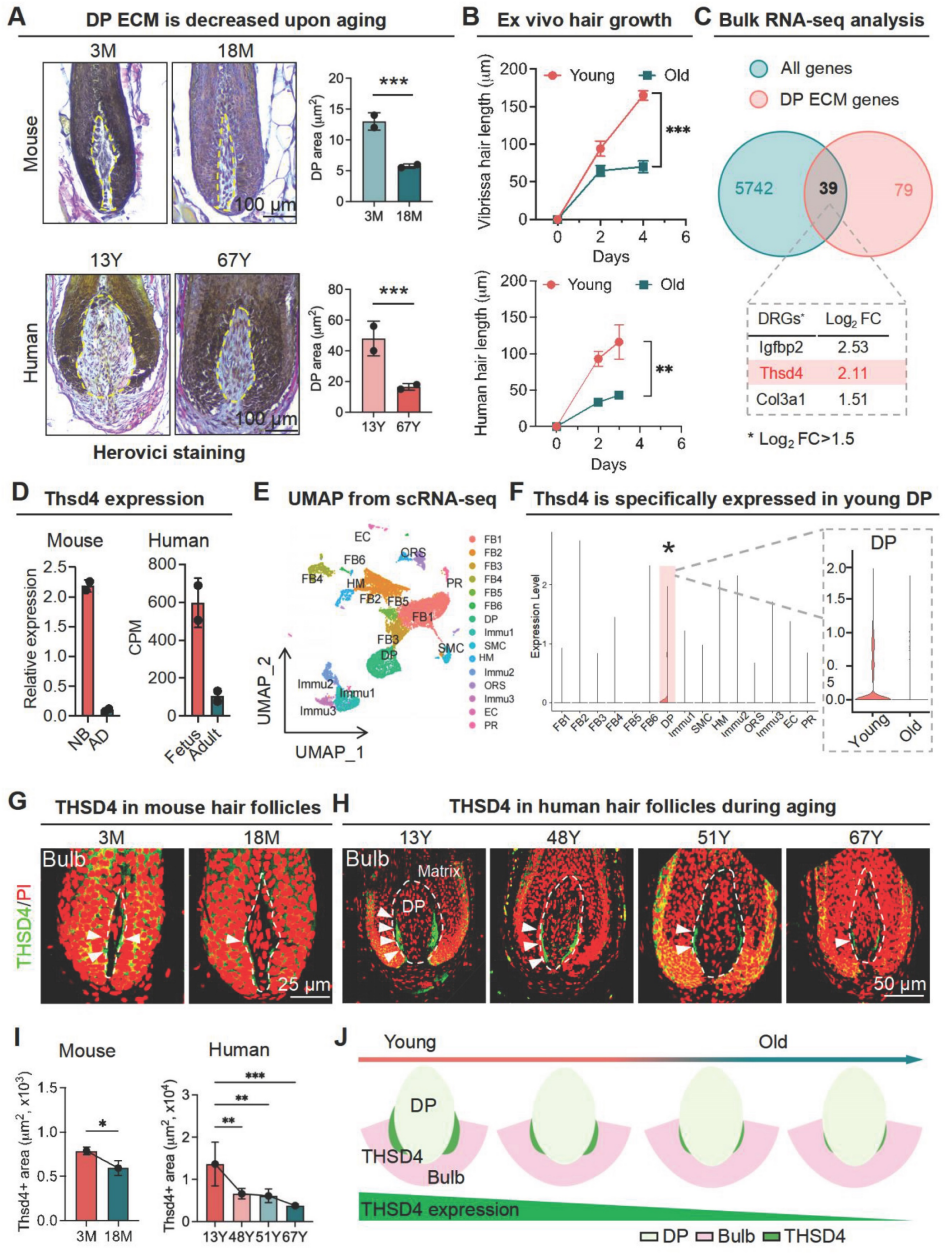
** THSD4 expression decreases upon aging. A.** Herovici staining of follicular bulge region showing maturation of DP matrix, quantification of DP area, and schematic representation of DP region and matrix content change during aging. N = 2, *** p < 0.001. scale bar = 100 μm. **B.** Hair shaft growth from cultured *ex vivo* hair follicles. N = 3, *** p < 0.001, ** p < 0.01. **C.** Venn diagram showing 39 differentially regulated genes that are related to DP ECM and the top 3 highest ranked genes according to their log_2_ fold change (Log_2_FC). **D.** Expression of Thsd4 mRNA in young and old mouse and human hair follicles by qRT-PCR or bulk RNA-seq, respectively. N = 2, CPM: counts per million. **E.** UMAP plot displaying different clusters of cell types of mouse skin. **F.** Vlnplot showing the mRNA expression of Thsd4 across various cell types of young and old skin. * and shaded area highlight the specific expression of THSD4 in DP. **G.** Immunofluorescence labeling of THSD4 (green) and PI (red) in murine vibrissal and human (H) scalp hair follicles. Representative images of bulb area and quantifications below show reduced Thsd4 levels during aging. Arrowheads show Thsd4 positive region. Scale bar (mouse) = 25 μm, scale bar (human) = 50 μm. **I.** Quantification of THSD4-positive area in hair bulb. N ≥ 3, *** p < 0.001, ** p < 0.01, and * p < 0.05. **J.** Schematic representation of THSD4 localization and expression change during aging.

During aging, HFSCs in the hair follicle bulge become quiescent partly due to the alteration of ECM changes that result in increased basement membrane stiffness, which causes hair follicle shrinking and hair loss eventually 26. Yet, given its indispensable role in the hair cycle and growth as the HFSC niche, how ECM changes in DP affect DP function remains to be investigated. To this end, we initially compared the global collagen content in the hair bulbs of young and old mouse vibrissae and human scalp hair follicles. Herovici staining of the DP region of both young and old hair follicles appeared blue, indicating mostly immature collagen with no differences in maturity. However, the internal ECM was widely distributed in young follicles and significantly reduced in aged follicles (Figure 1A, S1A-B). We then sought to verify whether the change in the DP content affected the growth capacity of these hair follicles *in vitro*. Indeed, the aging hair follicles with smaller DP areas and collagen content showed significantly lower growth rates compared to the young ones (Figure 1B, S1C).

To elucidate the molecular underpinnings of the ECM alterations within the DP during hair follicle aging, we submitted young and old murine vibrissal hair follicles for bulk RNA-seq. A weighted gene co-expression network analysis was performed where we overlapped the differentially expressed genes in young and aging follicles with those associated with the DP ECM, which resulted in 39 gene hits with Igfbp2, Thsd4, and Col3a1 being ranked the highest according to log_2_ fold change (Figure 1C, S1D), which was further confirmed using RNA-seq data from mouse skin and human scalp hair follicles (Figure 1D) as well as by qRT-PCR and Western blotting (WB) analysis (Figure S1E-F). Thus, our findings suggest that THSD4 expression has strong implications for hair follicle aging.

As a further step toward understanding the expression profile of THSD4, we performed scRNA-seq analysis for young and aged skin samples. We uncovered 15 distinct cell clusters in both young and old skin using their characteristic gene makers (Figure 1E, S1G-H). Importantly, the expression of Thsd4 was specifically detected only in the DP cell cluster of young follicles as it became dramatically downregulated during aging (Figure 1F, S2A). This was unlike the other two gene hits Igfbp2 and Col3a1 which had a broad-spectrum expression profile (Figure S2B-D). IF staining of both human and mouse hair follicles confirmed the significant decline of THSD4 with aging. Importantly, THSD4 was detected in the lower regions of the DP-HM junction (Figure 1G-I, S2E-F), suggesting a potential functional role in mediating epithelial-mesenchymal interaction (EMI), which is crucial for hair growth 32. Although not specific to the DP region, the expression of Col3a1, another ECM component, was also decreased during aging (Figure S2C-D), further supporting the loss of DP ECM content during follicular aging.

### THSD4 promotes hair growth by inducing hair matrix cell proliferation

**Figure 2:**
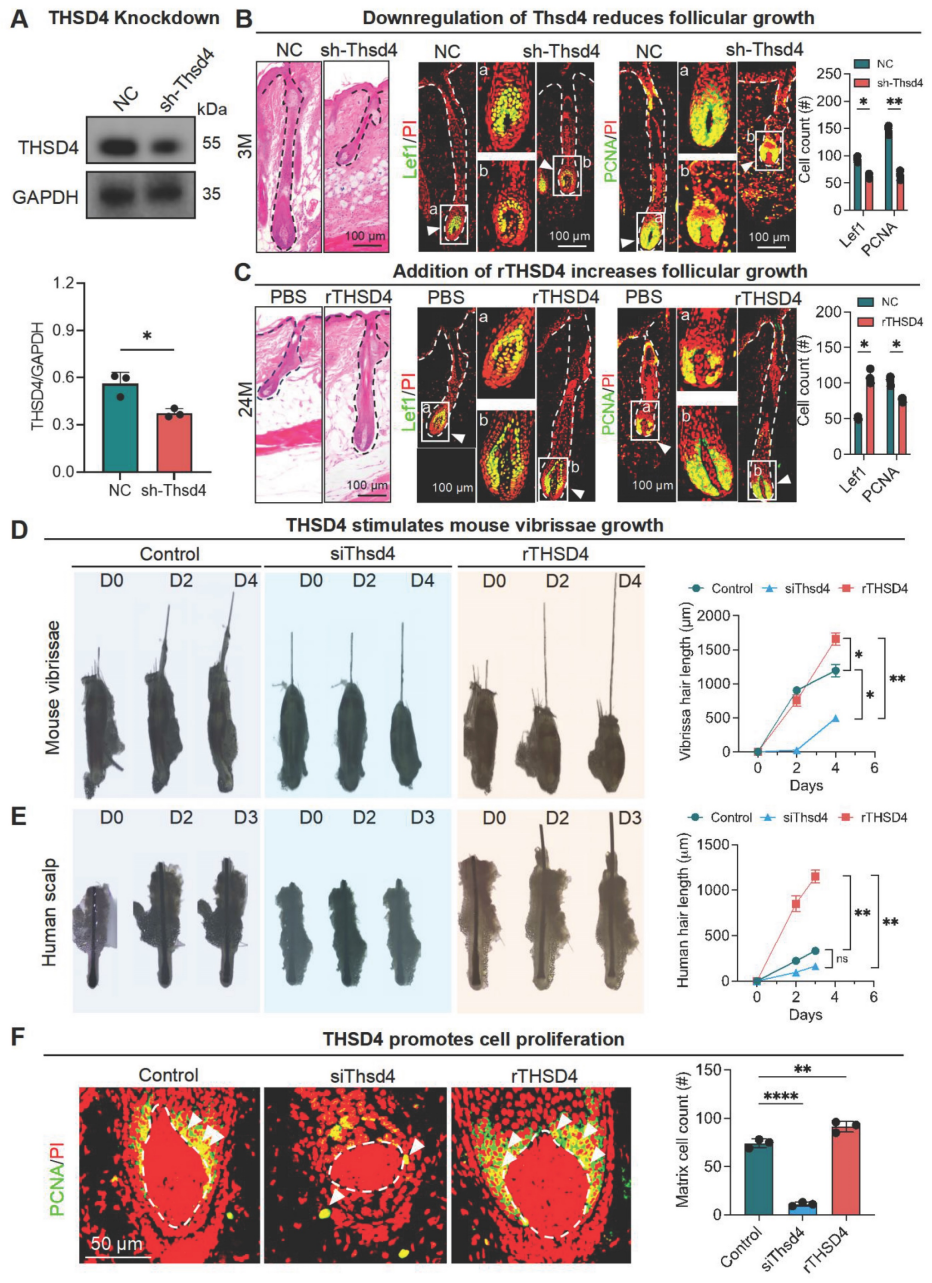
** THSD4 promotes hair generation by inducing DP cell proliferation. A.** Immunofluorescence images show successful delivery of Thsd4-targeting shRNA or its non-targeting control (NC), indicated by GFP fluorescence, via adenoviral infection of murine dorsal skin. Scale bar = 100 μm. **B.** Representative images of H&E staining and immunofluorescence labeling of Lef1 and PCNA of dorsal skin hair follicles of young (3M) mice with and without Thsd4 KD. Bar diagram to the right show reduced Lef1- and PCNA-positive cells after Thsd4 KD. N = 3, ** p < 0.01, and * p < 0.05. Scale bar = 100 μm. **C.** Representative images and quantification as described in **(B)** but of dorsal skin hair follicles of old (24M) mice. N = 3, * p < 0.05. Scale bar = 100 μm. **D.** Representative images showing hair growth from mouse vibrissal hair follicles with modulation of THSD4 levels, either by its siRNA or recombinant protein. N = 3, ** p < 0.01, and * p < 0.05. **E.** Representative images and quantification as depicted in **(D)** but of human scalp hair follicles. N = 3, ** p < 0.01, ns = not significant. **F.** Representative images of Immunofluorescence labeling of PCNA in mouse vibrissal hair follicles following manipulation of THSD4 levels. N = 3, **** p < 0.0001, and ** p < 0.01. Scale bar = 50 μm.

Next, we explored the functional role of THSD4 during hair follicle growth *in vivo*. Given the high expression of THSD4 in young hair follicles, we used lentiviral vectors to deliver Thsd4-targeting shRNAs to the dorsal skin hair follicles of young mice (3M) to assess its functional impact. GFP fluorescence indicates successful lentiviral infection and shRNA payload delivery, which led to a significant knockdown of THSD4 by western blotting (Figure 2A, S3A). In the control group, hair follicles reached the subcutaneous fat layer. However, hair follicles in the knockdown group were significantly shorter and remained in the early stage of the growth phase (Figure 2B, S3B-C). Thsd4 knockdown also reduced the number of Lef1- and PCNA-positive cells in the DP and hair matrix region compared to the control group (Figure 2B). On the other hand, we ectopically increased THSD4 levels in old mice (24M) by administering its recombinant protein (rTHSD4) into their dorsal skin. We found that supplementation of rTHSD4 led to increased follicular growth as well as Lef1- and PCNA-expressing of DP and matrix cells compared to those of the vehicle control group (Figure 2C, S3D). This suggests that THSD4 promotes hair follicle growth by regulating HM cell proliferation.

We then attempted to recapitulate the *in vivo* findings using *ex vivo* mouse vibrissal and human scalp and hair follicles in organ culture. Both mouse and human hair follicles showed similar morphology to those from the tissue specimens (Figure S3E). As expected, in both mouse (Figure 2D) and human (Figure 2E) *ex vivo* hair follicles, siRNA-mediated knockdown resulted in similar or shorter hair shafts, whereas treatment of rTHSD4 dramatically increased their length, compared to their respective controls. Similarly, IF staining showed a remarkable decrease in the number of PCNA-positive cells after Thsd4 knockdown and a significant increase in the number of those cells following rTHSD4 treatment in the DP and matrix of mouse vibrissal hair follicles (Figure 2F). Such changes in expression were also seen in Col III following knockdown or the addition of ectopic rTHSD4 in these hair follicles (Figure S3F). These results further suggest that DP-secreted THSD4 enhances hair follicle growth by promoting the proliferation of HM cells.

### THSD4 enhances the interaction between the epithelial and mesenchymal cells

**Figure 3:**
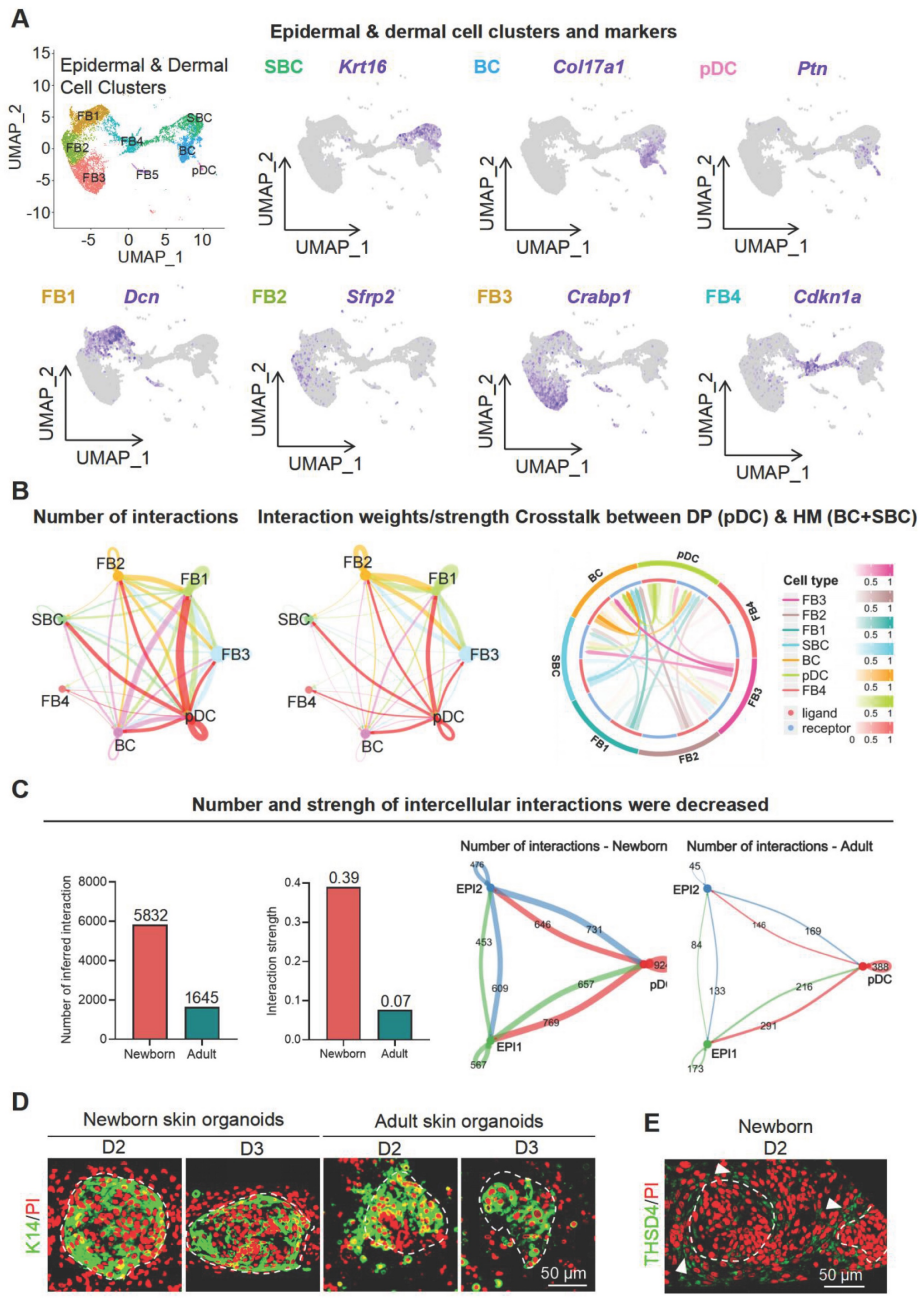
** ScRNA-seq analysis of skin organoids predicts epidermis-dermis interaction. A.** UMAP plots of epidermal and dermal clusters by unbiased clustering. **B.** CellChat and CellTalk analyses show potential interaction between DP (pDC) and HM (BC and SBC). **C.** CellChat analyses the strength of signaling interactions in neonatal and adult mice (Left) and interactions between epidermal and DP precursor cells are present in neonatal mice (Right). **D.** Immunostaining of K14 in skin organoids generated from newborn and adult mouse skin. Scale bar = 50 μm. **E.** Immunolabeling of Thsd4 in newborn mouse skin organoids. Arrowheads show Thsd4-positive (green) regions. Scale bar = 50 μm.

The growth of hair follicles is initiated by the interaction between epithelial and mesenchymal cells 33, 34. Our established organoid culture system serves as an excellent model for simulating epithelial-mesenchymal interactions, thus providing a platform for exploring the underlying cellular and molecular mechanisms 35-39. As we previously detected THSD4 in the HM (epithelium)-DP (mesenchyme) boundary, we isolated epidermal and dermal cells from scRNA-seq data of mouse skin organoids and predicted their interactions. We identified a total of 2 epithelial cell groups (SBC and BC) and 5 dermal cell groups (FB1-4 and pDC (putative DP precursors)) using the indicated specific gene markers (Figure 3A, S4A-B). Since skin organoids do not contain authentic DP cells, we used their precursor cells, pDC, for the downstream analyses. CellChat and CellCall analyses showed a notable number and strength of interactions between pDC and dermal cells (FB1-3) as well as SBC and BC groups which represent epithelial cells (Figure 3B), and that the number and strength of intercellular interactions decreased after aging (Figure 3C, S4C).

**Figure 4:**
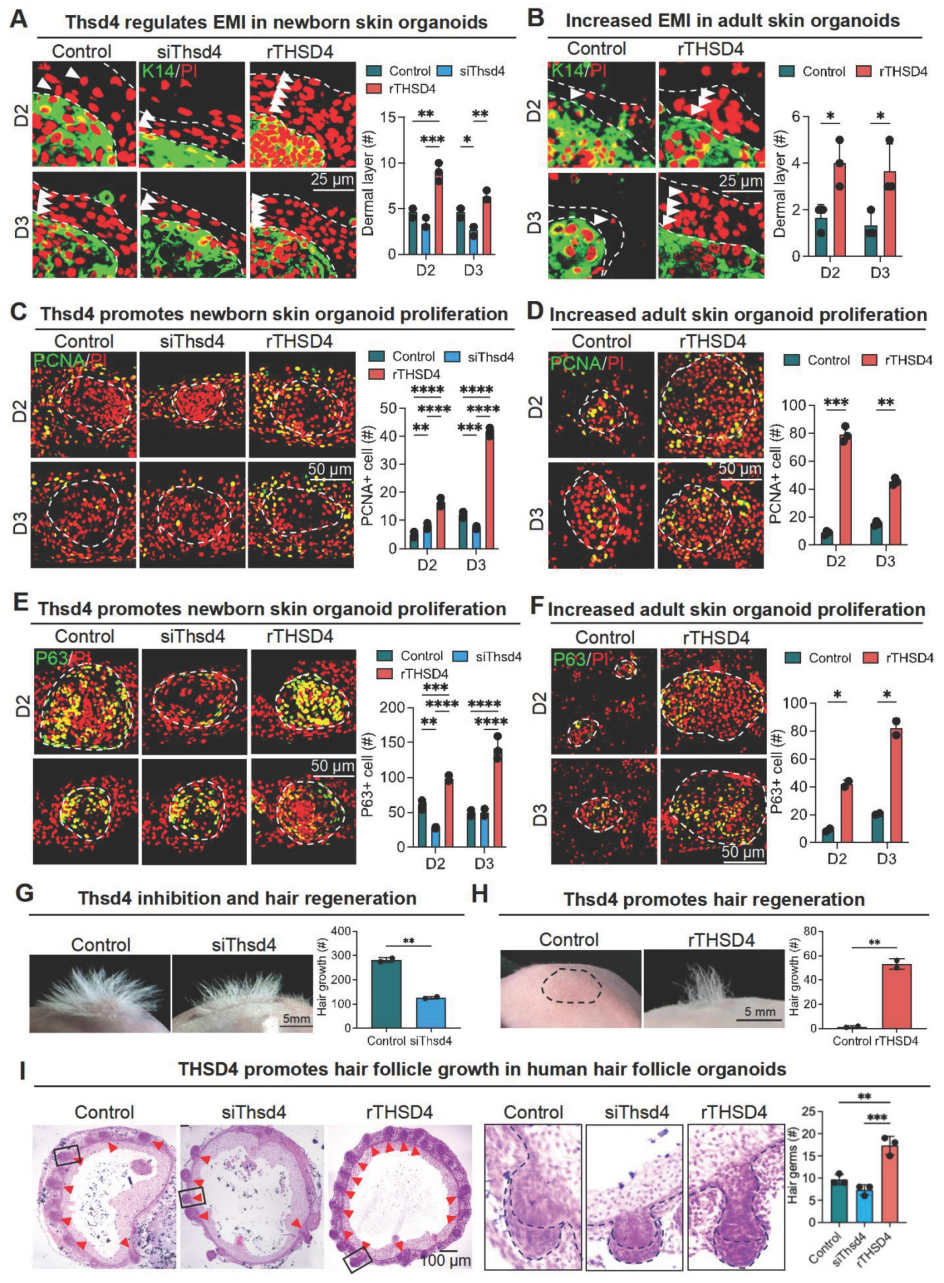
** THSD4 enhances adhesion between epidermis and dermis. A**. Immunofluorescence images and quantification showing dermal layer change (arrowheads) after treatment in newborn mouse skin organoids. N = 3, *** p < 0.001, ** p < 0.01, and * p < 0.05. Scale bar = 25 μm. **B.** Immunofluorescence images and quantification showing dermal layer change (arrowheads) after treatment in adult skin organoids. N = 3, * p < 0.05. Scale bar = 25 μm. **C.** Immunostaining of PCNA, and its quantification, of newborn mouse skin organoids after treatment. N = 3, **** p < 0.0001, *** p < 0.001, and ** p < 0.01. Scale bar = 25 μm. **D.** As described in **(C)** but in adult mouse skin organoids. N = 3, *** p < 0.001, and ** p < 0.01. Scale bar = 25 μm. **E.** Immunostaining of P63, and its quantification, of newborn mouse skin organoids after treatment. N = 3, **** p < 0.0001, *** p < 0.001, and ** p < 0.01. Scale bar = 25 μm. **F.** As described in **(E)** but in adult mouse skin organoids. N = 3, * p < 0.05. Scale bar = 25 μm. **G.** Representative images and quantification show reduction in hair generation from engrafted newborn mouse skin organoid culture after Thsd4 knockdown. N = 2, ** p < 0.01. Scale bar = 5 mm. **H.** Representative mages and quantification show increased hair generation from engrafted adult mouse skin organoid culture treated with recombinant THSD4 protein. N = 2, ** p < 0.01. Scale bar = 5 mm. **I.** H&E Staining shows the morphology (Left) and quantitative analysis of human hair follicle organoids. N = 3, ** p < 0.01, * p < 0.05, Scale bar = 100 μm.

Next, we used skin organoid culture for further validation. The epidermal cell aggregates in neonatal skin organoids appeared larger and more compact, with increased peripheral adhesion of dermal cells, whereas those in adult skin organoids appeared smaller, with fewer and scattered peripheral dermal cells (Figure 3D). Notably, THSD4 expression was detected in newborn skin organoids (Figure 3E), particularly in the dermal cells adjacent to the epidermal aggregate, and these dermal cells were identified as the putative DP precursors 36. This indicates that skin organoids can be used as an *in vitro* model to study the functional role of THSD4. Thus, we monitored skin organoid cell behavior after the modulation of THSD4 levels. In newborn skin organoids, THSD4 knockdown significantly reduced the number of layers of dermal cells surrounding the epidermis (Figure 4A). However, the opposite effect was observed in adult skin organoids upon treatment of exogenous rTHSD4 (Figure 4B, S5A-D). As THSD4 expression was decreased in aging hair follicles, we treated the adult skin organoids with rTHSD4 and observed a notable increase in the aggregate size (Figure S5E) and a marked increase in the number of dermal cell layers surrounding the epidermal aggregates (Figure 4B). These results suggest that THSD4 facilitates the interaction between the epidermal and dermal cells, which aligns with our previous observation.

Next, we assessed epidermal cell proliferation and stemness using PCNA (Figure 4C-D) and P63 (Figure 4E-F) as their respective markers following THSD4 modulation in the newborn (Figure 4E-G) and adult (Figure 4F-H) skin organoids. Similar to our previous findings, THSD4 knockdown led to a dramatic reduction in the number of PCNA- and P63-positive cells in newborn skin organoids (Figure 4C, E), but rTHSD4 treatment significantly increased their numbers in both newborn and adult skin organoids compared to their respective controls (Figure 4C-F). Importantly, nude mice transplanted with newborn skin organoids with THSD4 knockdown produced significantly fewer hairs compared to the control (Figure 4G), whereas rTHSD4 treatment in adult skin organoids generated significantly more hairs compared to the control after transplantation (Figure 4H). Importantly, rTHSD4 also increased the number of follicles formed in human induced pluripotent stem cell-derived skin organoids, which remarkably recapitulated our results from the murine models (Figure 4I, S6A-B). Together, our results suggest a potential role of THSD4 in regulating epithelial-mesenchymal interaction, which is required for hair growth in young and aging hair follicles.

### SDC4 induces THSD4 expression to promote hair growth

**Figure 5:**
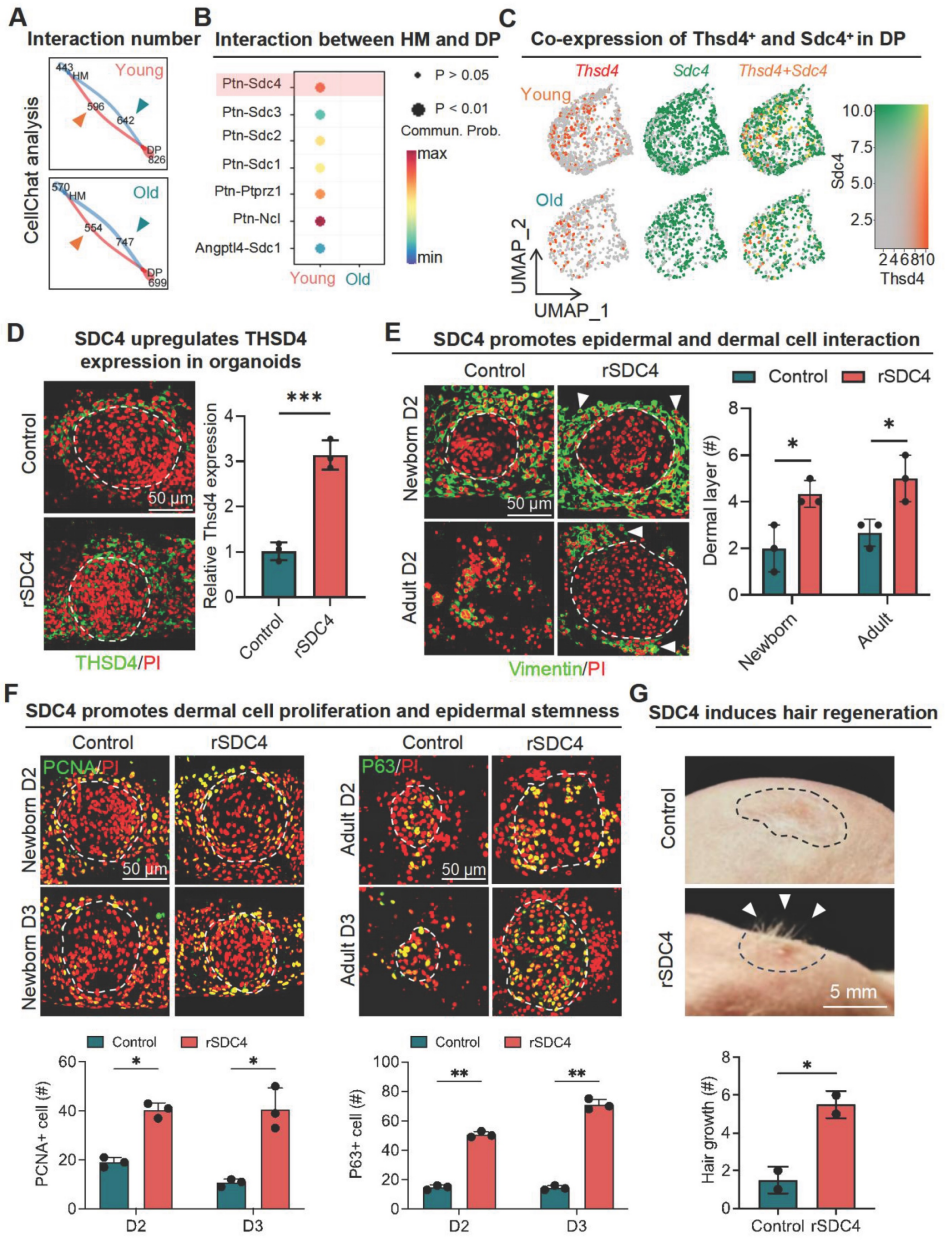
** Interaction between hair matrix and DP drives THSD4 expression via SDC4. A.** Chord diagram shows number and strength of interactions between DP and hair matrix (HM) in young and old murine dorsal hair follicles. **B.** CellChat analysis shows enriched interaction between hair DP and HM via Ptn-Sdc4 signaling in young murine hair follicles. **C.** Feature plots show co-expression of Thsd4 and Sdc4 in DP cell cluster in young and old mouse hair follicles. **D.** Immunofluorescence image of newborn mouse skin organoids shows increased THSD4 expression after treatment with recombinant SDC4 protein. Scale bar = 50 μm. **E.** Immunofluorescence images and quantification of newborn and adult mouse skin organoids show increased adhesion of dermal cells around epidermal cells after recombinant SDC4 protein treatment. N = 3, * p < 0.05. Scale bar = 50 μm. **F.** PCNA and P63 immunostaining, and their quantification below, show increased proliferation and stemness in newborn and adult mouse skin organoids, respectively. N = 3, ** p < 0.01, and * p < 0.05. Scale bar = 50 μm. **G.** Representative mages and quantification show increased hair generation from engrafted adult mouse skin organoid culture treated with recombinant SDC4 protein. N = 2, * p < 0.05. Scale bar = 5 mm.

To identify potential upstream regulators of THSD4 in DP, we performed CellChat analysis again using scRNA-seq data from young and old mouse skin hair follicles. We focused our analysis on the HM and DP compartments as they were previously shown to potentially interact. Indeed, we found that HM-DP interaction was stronger in young hair follicles compared to the old (Figure 5A, S7A) and signaling Ptn (HM) to Sdc4 (DP) was amongst the most prominent ligand/receptor pairs enriched only in the young hair follicles (Figure 5B). More importantly, Sdc4 exhibited a similar pattern of expression to that of Thsd4 such that it was highly expressed in the DP of young hair follicles but became noticeably downregulated upon aging (Figure 5C, S7B).

In corroboration with our *in-silico* analysis, the addition of recombinant SDC4 (rSDC4) in newborn skin organoid cells upregulated THSD4 expression (Figure 5D, S7C) and increased the number of dermal cell layers adhering to the epidermis in both newborn and adult skin organoids (Figure 5E, S7D-E), suggesting enhanced epithelial-mesenchymal interaction as seen previously. A larger number of PCNA- and P63-positive cells were also detected in both newborn and adult skin organoids, indicating increased proliferative capacity (Figure 5F). Subsequent *in vivo* study showed that treatment of rSDC4 alone resulted in significant hair regeneration from adult skin organoids after transplantation in nude mice (Figure 5G). Collectively, these results suggest that SDC4 likely mediates hair regeneration through upregulation of THSD4 in the DP of hair follicles.

### THSD4 stimulates hair growth via upregulation of CXCL1

**Figure 6:**
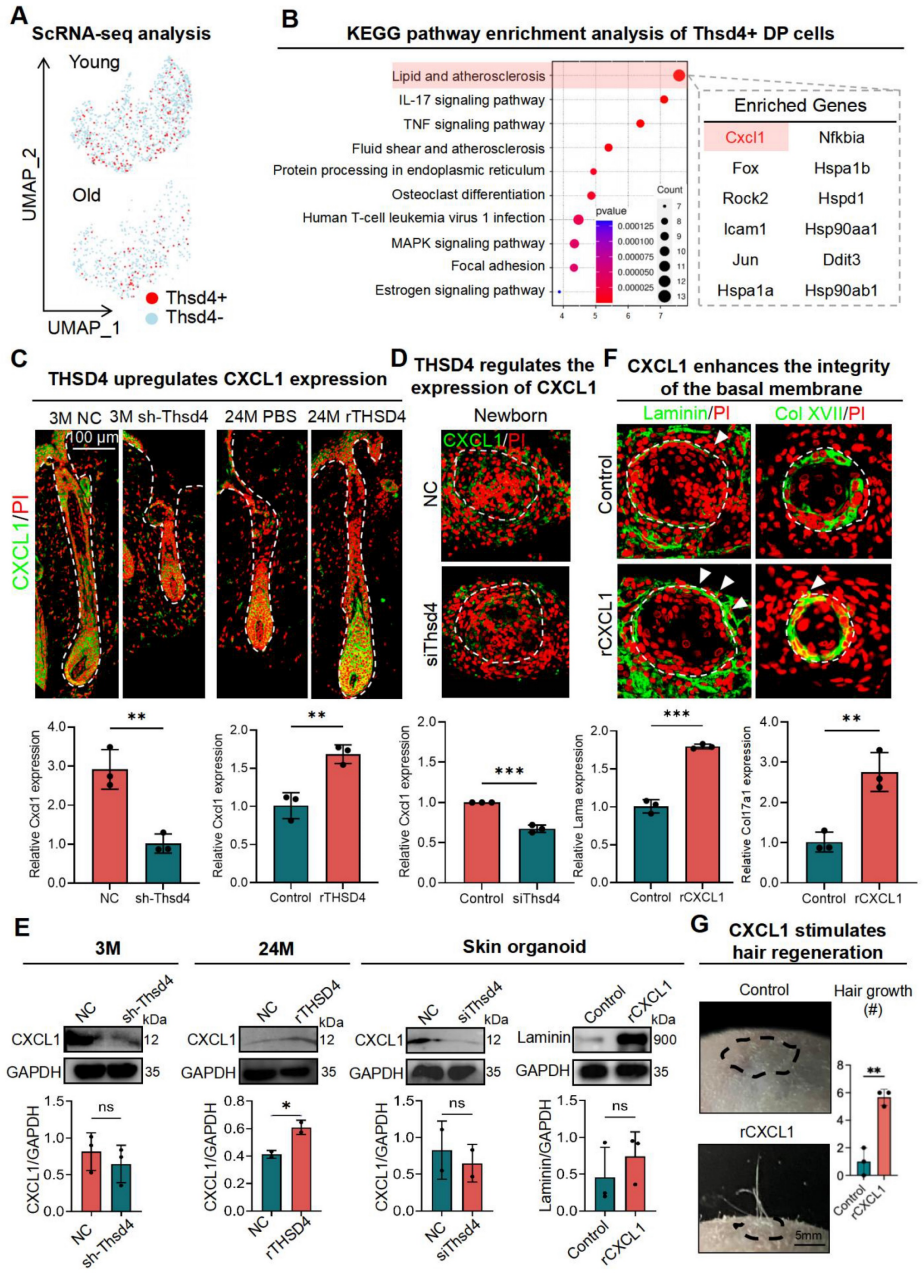
** THSD4 stimulates hair generation via upregulation of CXCL1. A.** Feature plots show reduced Thsd4-expressing cells in the DP cell cluster in old mouse hair follicles. **B.** KEGG pathway enrichment analysis of the bulk RNA-seq data and the list of enriched genes within the top-ranked pathway. **C.** Immunofluorescence images, and their qRT-PCR quantifications, of Cxcl1 in young (3M) and old (24M) mouse vibrissal hair follicles following shRNA-mediated KD of Thsd4 or recombinant Thsd4 protein treatment, respectively. N = 3, ** p < 0.01, Scale bar = 100 μm. **D.** Immunostaining of CXCL1 and its qRT-PCR quantification in newborn mouse skin organoids show reduced CXCL1 expression after treatment with Thsd4-targeting siRNA compared to the controls (NC). N = 3, *** p < 0.001. Scale bar = 50 μm. **E.** Western blotting analyses showing CXCL1 and Laminin expression after RNA interference or recombinant protein treatments. N = 3, * p < 0.05, ns = no significance. **F.** Immunolabeling of Laminin and Col XVII in newborn mouse skin organoids show enhanced basal membrane integrity, as quantified by qRT-PCR below, after treatment with recombinant CXCL1. N = 3, ** p < 0.01, *** p < 0.001. Scale bar = 50 μm. **G.** Representative images and quantification show increased hair generation from engrafted adult mouse skin organoid culture after treatment with recombinant Cxcl1. N = 3, ** p < 0.01. Scale bar = 5 mm.

We next sought to identify the downstream targets of THSD4. Using the scRNA-seq data, we first identified the Thsd4-positive cells in the DP of young and aged hair follicles (Figure 6A) and then subjected them to KEGG pathway analysis, which resulted in distinct enrichment profiles based on aging (Figure 6B, S8A). Due to the higher expression of THSD4, we focused on the pathways from the young hair follicle in which lipid metabolism and atherosclerosis were ranked the highest (Figure 6B). Within this pathway, Cxcl1, encoding chemokine (C-X-C motif) ligand 1, was one of the candidate genes (Figure 6B). Given its high expression in young hair follicles and prominent role in promoting cell proliferation and migration 40, we explored Cxcl1 further.

To this end, we first assessed the change in CXCL1 levels in the dorsal skin of mice after modulation of THSD4. Immunofluorescence labeling of mouse dorsal skin hair follicle samples showed downregulation of Cxcl1 after Thsd4 silencing and its upregulation following treatment of rTHSD4, particularly at the HM-DP junction region (Figure 6C). This was also recapitulated in newborn skin organoids, in which the addition of rTHSD4 results in increased CXCL1 expression in the dermal cells surrounding the epidermal aggregates, compared to the control (Figure 6D-E). Furthermore, the addition of exogenous CXCL1 (rCXCL1) in adult skin organoids led to improved basal membrane integrity evidenced by the increased Laminin (Lama) and Col XVII (Col17a1) expression around the boundary of the dermis and epidermis (Figure 6F), indicating increased epithelial-mesenchymal interaction. Once more, we observed significant hair growth from rCXCL1-treated adult cell skin organoids after transplantation into adult mice, whereas no hair growth was seen in the control mice (Figure 6G). These findings demonstrate that THSD4 promotes hair growth, at least in part, through the upregulation of Cxcl1.

### Exposure to low temperature promotes hair growth in aging follicles

**Figure 7:**
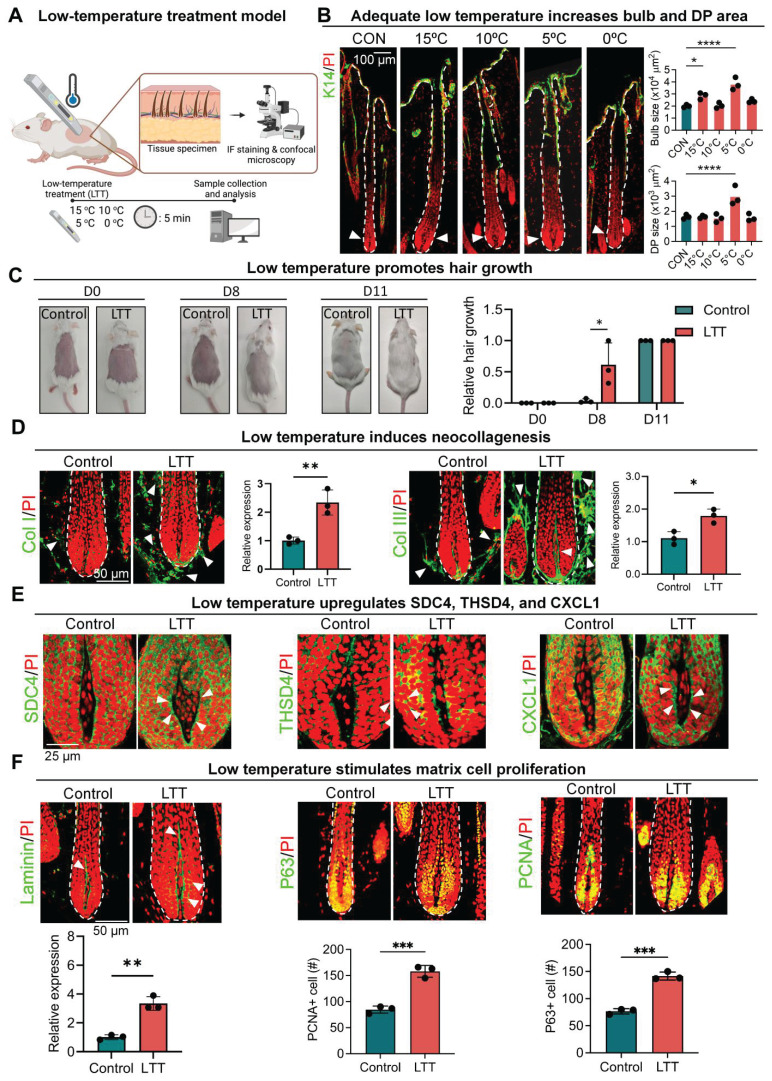
** Low temperature promotes hair growth. A.** Graphical illustration of the effects of temperature on hair growth. **B.** Immunofluorescence labeling of K14 in murine dorsal skin hair follicles after 5-minute exposure to various low temperatures and quantification of the hair bulb and DP size. N = 3, **** p < 0.0001, * p < 0.05, scale bar = 100 μm. **C.** Low temperatures stimulate hair growth in mice. N = 4, * p < 0.05, scale bar = 50 μm. **D.** Representative immunofluorescence images and quantification show increased Col I and Col III expression after low temperature treatment (LTT) at 5 ^o^C for 5 minutes. Arrowheads indicated Col I- or Col III-positive regions. N = 3, ** p < 0.01, * p < 0.05. Scale bar = 50 μm. **E.** Representative immunofluorescence images and quantification show increased SDC4, THSD4, and CXCL1 expression after LTT. Arrowheads indicated SDC4-, THSD4-, or CXCL1-positive regions in the DP-HM interface. N = 3, * p < 0.05. Scale bar = 25 μm. **F.** Representative immunofluorescence images and quantification show increased Laminin, P63, and PCNA expression after LTT. Arrowheads indicated Laminin-positive region. N = 3, *** p < 0.001. Scale bar = 50 μm.

So far, our results indicate that follicular micro-environment changes can be transmitted to the DP via THSD4-mediated direct or reciprocal interaction between the epithelium and mesenchyme to influence hair growth. As various external environmental factors can also alter follicular function and hair growth 14, 41, we wondered whether they influence hair growth via this newly found mechanism. Interestingly, exposure to non-freezing low temperatures can effectively alter the physiology of human tissues by modification of ECM 42, 43. We then used low temperature as the representative macro-environmental stimulus for our subsequent investigations. But first, to explore how low temperature affects hair growth, we transiently exposed aging follicles from 9-month-old mice to various low temperatures (15 ºC - 0 ºC) for 5 min (Figure 7A). Surprisingly, we found treatment at 5 ºC, referred to as low-temperature treatment (LTT) from here onwards, led to significant increases in hair bulb size and DP area (Figure 7B-C, S9A), suggesting potential enhancement in hair growth. Notably, transient exposure to low temperatures also induced the expression of type I and III collagen (Figure 7D), the two most common types of collagen in the skin as expected, which corroborated with previous reports. More importantly, the expression of SDC4, THSD4, and CXCL1 was also significantly upregulated simultaneously with this increase in the collagen deposition around the hair follicle (Figure 7E, S9B-C). This suggests that the SDC4/THSD4/CXCL1 axis was part of the sensory response mechanism that transmits the external macro-environmental signals to the, DP, the master regulator of hair growth. Indeed, further IF and WB analyses showed that this low-temperature treatment significantly increased the expression of THSD4 (Figure 7E, S9B-C), which was indicative of increased DP-HM interaction, as well as both P63- and PCNA-positive cells in the HM, signifying enhanced hair growth (Figure 7F). Altogether, these results demonstrate for the first time the effect of non-freezing low temperatures on hair growth and implicate THSD4 in relaying physical cues from the external macro-environment to the DP, further extending its functional importance.

## Discussion

Increased degradation of ECM-related proteins in aging skin tissue leads to a reduced connection between collagen fibers and dermal cells, and may disrupt the organization and behavior of dermal cells 44, 45. These changes in the dermal ECM have a direct impact on the micro-environment surrounding hair follicles resulting in disruption of the hair growth cycles and their untimely degeneration 26, 46. However, how aging-related changes in DP ECM influence hair growth has been seldom investigated. In this present study, we investigated the changes in the DP ECM during aging and how it contributed to hair follicular degeneration. We report here that DP ECM undergoes dramatic reduction during aging, including a critical component, THSD4, which is specifically detected in the DP close to the outermost border of the HM. THSD4 promotes hair growth in aged follicles by regulating EMI between DP and HM via SDC4 and CXCL1. Using low temperature as a stimulus, we further implicate this novel mechanism in propagating signals from the external macro-environment to the hair follicle DP to modulate hair growth.

One particularly interesting finding is that, in addition to dermal ECM, the DP ECM also undergoes remarkable depletion during aging, including THSD4, an ECM component expressed in the interface between DP and HM. THSD4 (Thrombospondin type-1 domain-containing 4) encodes ADAMTSL6 (A disintegrin and metalloproteinase with thrombospondin motif-like protein 6), which belongs to the ADAMTS family of extracellular metalloproteinases involved in enzymatic modification of ECM 47. However, ADAMTS-like (ADAMTSL) proteins such as ADAMSTL6 are secreted glycoproteins without enzymatic activity 48. Instead, ADAMTSL6 (denoted as THSD4 for consistency from here on) is known to physically interact with fibrillin-1 to induce Fibrillin-1 matrix assembly into microfibrils, and loss of function mutation in Thsd4 results in impaired fibrillin-1 microfibril assembly in human patient specimens 49. Fibrillin-1 is an important building block of the elastic network in the dermal ECM, which endows important biomechanical properties 50. As the skin undergoes age-induced remodeling of the ECM, which includes changes in the elastin and microfibril content, the levels of THSD4 are likely to also be affected. In addition, fibrillin-1 is found in the dermal-epidermal junction of the skin which provides adhesion to the basement membrane at the dermal-epidermal junction 51. Mutations in the fibrillin-1 gene, FBN1, have been reported to cause defects in the ECM thus weakening the supporting tissues 52. Consistently, we observed increased adhesion between the epidermis and dermis in our organotypic cell cultures after the addition of exogenous THSD4, and that Thsd4 knockdown resulted in impaired integrity of the basal membrane.

More importantly, the drastic changes in the DP ECM may alter its surrounding micro-environment, and vice versa, thus influencing the mesenchyme to epithelial interactions critical for regulation of hair growth during aging. Indeed, our bioinformatic analyses and subsequent functional validations provide strong evidence that DP cells can be modulated by hair matrix. Specifically, we identified SDC4, likely under regulation by molecular signals from the HM, that regulates THSD4 expression in the DP cells. SDC4, or Syndecan 4, is a ubiquitously expressed cell surface-residing proteoglycan 53. SDC4 can act as co-receptors for FGF receptor (FGFR) to prolong and strengthen the signals, yet it can function independently as a receptor for FGF, vascular endothelial growth factor (VEGF), and platelet-derived growth factors (PDGF) to initiate respective signaling cascades which are important for hair development, growth and cycle. However, our evidence showing THSD4 induction by exogenous SDC4 suggests that the matrix cells can influence hair growth by transmitting this signaling cascade in a paracrine ligand-receptor fashion. As SDC4 can directly interact with the ECM and initiate signal transduction into the cytosol through the cytoskeleton, it may upregulate THSD4 in this fashion 54. Interestingly, SDC4 and THSD4 are co-expressed in the DP, thus upregulation of SDC4 in DP cells may help potentiate the signals from the hair matrix in a feed-forward loop as the increased THSD4 further strengthens the interaction between the dermal and epidermal cells. However, this exact mechanism requires further investigation. Nevertheless, the ECM has been implicated in the regulation of the activity and function of the chemokine CXCL1 55, 56. Some ECM components can bind and stabilize CXCL1, thereby enhancing its biological activity. For instance, cell surface proteoglycans play a crucial role in cell adhesion and act as receptors for growth factors, enzymes, and chemokines 56. Furthermore, the epidermal growth factor can act as a carrier for CXCL1, altering its diffusion and concentration gradient in the extracellular space, affecting cellular responses, and ultimately hair growth. Studies have demonstrated that vascularization is essential for promoting hair growth in neonates 57, and CXCL1 plays a crucial role in the processes of angiogenesis and trauma repair, which are closely related to hair follicle growth 43, 58-60. Increases in CXCL1 secretion have been associated with the upregulation of key hair development genes such as Wnt5a, Wnt10b, and LEF1, which are essential to hair follicle growth. Additionally, other studies have reported that Minoxidil enhances the expression of CXCL1, which, in turn, increases the proliferative capacity of DP cells and elongates hair shafts in organoid culture models 61. These findings collectively underscore the pivotal role of CXCL1 in hair follicle development and growth, supporting its potential use as a therapeutic target for hair regeneration and repair.

Notably, our finding can be seamlessly translated in the clinical setting as this reciprocal molecular signaling cascade between mesenchymal and epithelial cells can respond to macro-environmental factors as such a drop in ambient temperature. Studies have demonstrated the effect of physiologically relevant temperatures on the synthesis, structural change and stability of collagens. Application of low temperature at 18 ºC can stimulate the deposition of collagens in the muscle zebra fish and structural remodeling of human lung type I collagen can only be observed at a lower temperature of 30 ºC 43, 62. As the major component of skin ECM, alteration of collagens may reflect the macro-environmental changes in the DP micro-environment. Moreover, exposure to cold temperatures has various effects on the skin as a whole such as facilitating wound healing, activating skin cell metabolism, and modulating molecular profiles such as the production of reactive oxygen species (ROS) and nitric oxide (NO) 55. Thus, temperature treatment, especially the use of non-freezing low temperatures, as opposed to cryotherapy, is increasingly being implemented in both clinical and non-clinical settings. For example, low-temperature treatments are used to treat alopecia areata and atopic dermatitis owing to their anti-inflammatory effects 63-65. Our results suggest that the application of adequate low temperature (5 ºC) can remodel the ECM components of hair follicles, such as type I and III collagens and particularly THSD4. As type I collagen is the most abundant collagen in youthful skin, which serves as an unambiguous marker for skin aging 66, low-temperature treatment may help delay the aging process of the skin and its appendages. In addition to the existing evidence, our findings suggest a new role of low temperatures in facilitating epithelium and mesenchyme interaction by reshaping the ECM of DP and HM, thus modulating hair growth.

In summary, our results underline the importance of maintaining THSD4 expression in DP cells for anti-aging and enhanced hair-inducing effects. Moreover, our study sheds new light on the mechanism that leads to the aggregation of DP cell populations and the crosstalk between dermal and epidermal cells during aging. Nonetheless, the results herein constitute the first evidence demonstrating the hair-promoting effects of non-freezing low-temperature treatment, which opens an avenue for its potential application in the clinic.

## Materials and methods

### Animals and tissue samples

Human scalp samples, CD1 mice, and nude mice were used in this study. 3-month-old (30), 6-month-old (6), 9-month-old (9), 18-month-old (6), 24-month-old (6) and 2-month-oldnude mice (24) of the CD1 (ICR) strain were obtained from the Beijing Viton Lever Laboratory Animal Technology Co., Ltd. The mice were kept in an animal room at a temperature of 25 °C, with adequate water and food, 12 h of light per day, and bedding changed every 3 days, and the mice were in good condition to meet the experimental requirements. Human scalp follicles were obtained from the Central Theatre General Hospital, and human scalp samples were collected from 13, 48, 57, and 68 years of age. There were 6 paraffin samples was 6, and each paraffin block contained several hair follicles. All procedures were performed in accordance with the Institutional Animal Care and Use Committees of Chongqing University.

### Tissue culture conditions

*Ex vivo* human hair follicles were maintained in William'E medium (#A1217601, Merck, Germany ) supplemented with 1% Fetal bovine serum (#10099-141C, Gibco, USA), 2 mM Glutamine (#G8230-25g, Solarbio, China), 2 mM Hepes (#H8090-25g, Solarbio, China), 10 ng/mL Hydrocortisone (#50-23-7, MCE, China), 10 μg/mL Bovine insulin (#18040-50mg, Solarbio, China), 10 μg/mL Transferrin (#T3309, Sigma, USA), 10 ng/mL Sodium selenite (#S5261-10g, Sigma, USA), 100 U/mL Penicillin (#15140-122, Gibco, USA), 100 μg/mL Streptomycin (#15140-122, Gibco, USA), and 2.5 μg/mL Amphotericin B (#A8250, Solarbio, China). Mouse vibrissa hair follicles and primary cell culture were maintained in DMEM/F12 medium (#MT10013CV, Corning, USA) supplemented with 10% fetal bovine serum (#10099-141C, Gibco, USA), 100 U/mL Penicillin, and 100 μg/mL Streptomycin. All tissue cultures were incubated at 37 °C and 5% CO_2_ unless mentioned otherwise.

### *Ex vivo* culture of hair follicles

The human scalp tissue samples were obtained from the Central Theatre Army General Hospital, Wuhan, China, after institutional ethics approval. To isolate hair follicles, a longitudinal excision was first made on the scalp tissue using a sterile scalpel to remove a row of tissue containing hair follicles. Individual hair follicles were then removed from the tissue by surgical excisions along the direction of its growth. The separated hair follicles in the anagen phase were further identified and selected for analysis. The vibrissal hair follicles were isolated from day 7 (young) and 2 (adult) month-old CD1 mice. The whisker hair was first trimmed before small skin samples were surgically removed. The tentacle hair follicles were separated by gently scratching the epidermis of the tentacle hair follicles at the junction with the skin epidermis using a 1-ml sterile hypodermic needle. The isolated hair follicles from both human scalp and mouse vibrissae were placed in their respective culture medium to recover for 24 h before recording hair growth in the indicated days using a light microscope. All procedures were approved by the Institutional Animal Care and Use Committees at Chongqing University.

### Primary cell culture

The mouse skin organoid culture was generated as described before 37. The dorsal skin of neonatal and adult mice was separated into epidermis and dermis and dissociated into single-cell cultures after digestion with trypsin and collagenase I. The epidermal and dermal cells were mixed at a ratio of 1:9, washed with BI serum (#04-001-1ACS, Biological Industries, China), and then placed into a Transwell inside a 12-well plate allowing the formation of an air-liquid interface. The cells were cultured in DMEM/F12 supplemented with 10% fetal bovine serum and 1% penicillin/streptomycin. The culture medium was refreshed every two days.

### Human hair follicle organoid procurement

Human embryonic stem cells (hESCs; WA25 cell line) were separated into single cells and plated on 96-well U-bottom plates to create uniform cell aggregates. We then transferred the aggregates to new plates containing differentiation medium to promote epidermal differentiation as previously described 67, 68. When the hair buds began to form, recombinant THSD4 (70 ng/mL and 200 ng/mL) or siRNA targeting Thsd4 (50 nM and 150 nM) were added for 7 days prior to analysis by H&E staining.

### RNA interference

Vibrissal hair follicles in anagen were transfected with Thsd4-targeting siRNA (5' GACUGUGUCCCUGAAGUUGAU 3') using a commercial siRNA reagent system (Beijing Tsingke Biotech Co., Ltd., China) following the manufacturer's instructions 69-71. Briefly, stock solutions (10 µM) of Thsd4 siRNA and its non-targeting control were prepared using RNAse-free water. After micromanipulation to isolate mouse antennal hair follicles, the follicles were equilibrated with fresh William'E medium (#A1217601, Merck, Germany) for 24 h. Hair follicles were transfected 24 h after 6 h of microdissection using 100 nM siRNA. Finally, fresh William'E medium was replaced every second day and after 5 days of culture. For *in vivo* knockdown, lentiviruses carrying Thsd4-targeting shRNA (5' GACTGTGTCCCTGAAGTTGAT 3') or its non-targeting control were purchased from GenePharma (GenePharma, Shanghai, China). Two days after plucking, lentiviruses were administered at a titer of 1x10^8^ UT in 40 μL to the dorsal skin the CD1 mice every other day for a total of 3 injections. Then the dorsal skin was surgically removed for subsequent analyses.

### Transplantation

Primary dermal/epidermal cell cultures of different treatments were grafted to the dorsal skin of nude mice using our previously described planar hair forming assay to assess the hair regenerative capacity 37, 72. Briefly, a small wound area equal to the size of the engrafting cell mixture was created on the dorsal skin of nude mice. The cell mixture on the Transwell membrane was flipped onto the wound with the cell size down. The membrane was then sutured onto the dorsal skin of the host. The wound area was bandaged after the application of sterile dressings. Two weeks after transplantation, the bandage was removed, and the regenerated hair was photographed and counted.

### Immunofluorescence

The formalin-fixed and paraffin-embedded sections were dewaxed, rehydrated, and antigen repaired using citric acid (#C805019, Macklin, China) and sodium citrate (#S818273, Macklin, China) solution. The samples were blocked with 2% bovine serum albumin (BSA; #A8020, Solarbio, China) before overnight incubation with primary antibodies at 4 °C and subsequent labeling of fluorochrome-conjugated secondary antibodies for 1 h at room temperature. Glass slides were rinsed with PBS and sealed with an anti-fluorescent quenching agent (#S2100, Solarbio, China). Images were taken using a Leica Cell Viewing System (Leica, Germany) with a 10X objective.

### Hematoxylin eosin and herovici staining

For Hematoxylin Eosin and Herovici Staining, the rehydrated sections were stained either with hematoxylin & eosin solution (#G1120, Solarbio, China) for 1-2 min or Herovici dye (HSK, Scytek, China) for 2 min before being destained, dehydrated, and sealed with cover glasses. The sections were viewed and photographed onto neutral gum-based films using a camera-mounted microscope.

### Quantitative RT-PCR

Total RNA from the skin sample was extracted using Trizol (NR0002, Regen, China) according to the manufacturer's instructions and reverse transcribed with reverse transcriptase (RT-01023, FOREG ENE, China). The synthesized cDNA was used as template for quantitative PCR using 2X SYBR Green q-RT PCR Master Mix (#B21203, Takara, Japan) on PCR Real-Time system with specific primers for target genes. The prime sequence in PCR is included in the supplementary table 1. The relative expression was determined using the “ΔΔCt” method 73.

### Western blotting

Total protein was extracted using RIPA buffer (# P0013D, Beyotime, China). The protein lysate concentration was determined using the BCA Protein Assay Kit (Beyotime, Haimen, China). Protein lysates were separated by 10% SDS-PAGE (Tengyi, Chongqing, China) and transferred to a PVDF membrane (#1620177, Tengyi, China). After 1 h at room temperature in 5% skimmed milk, the membranes were incubated overnight at 4 °C with the corresponding primary antibodies and 1 h at room temperature with horseradish peroxidase-coupled secondary antibodies. Dilute primary antibody 1000x and secondary antibody 2000x. Immunoreactive bands were imaged using an imaging system (Bio-Rad, USA).

### Bulk RNA-sequencing and analysis

Data on young and aged dermal papillae of follicles are from the cited article 74. The cryogenics data came from the sequencing of our own samples, including the dorsal skin of control and cryogenically treated mice. We collected skin samples from the control group 14 days after plucking and from the group treated with cryogenics 14 days after plucking. High-throughput transcriptome sequencing was performed on these skin samples. Library construction and sequencing were performed at Shanghai Majorbio Bio-pharm Biotechnology Co., Ltd. (Shanghai, China) according to the manufacturer's instructions (Illumina, San Diego, 177 CA). Bulk RNA-seq data were analyzed on the online Majorbio Cloud Platform (www.majorbio.com).

### ScRNA-sequencing analysis

The scRNA-seq data of mouse hair follicles (GSE115424) were obtained from the GEO database and re-analyzed 8. We used Seurat v.4.1.1 to perform QC, normalization, feature selection, linear and non-linear dimensional reduction, cell clustering by biomarkers, and cell cluster identify assignment. Cells with a mitochondrial content greater than 5% were excluded, and a dimensionality reduction by UMAP (Uniform Manifold Approximation and Projection) was performed for dimensionality reduction. This resulted in the identification of a total of 15 subpopulations, each of which was identified based on the marker genes that were specifically expressed in that subpopulation. The identity of each subpopulation was annotated using the CellMarker database, the Single R package, and prior knowledge in the field of hair follicle research. The cell subpopulation types include fibroblast 1 (Fibroblast 1, FB1), fibroblast 2 (Fibroblast 2, FB2), fibroblast 3 (Fibroblast 3, FB3), fibroblast 4 (FB4), fibroblast 5 (FB5), fibroblast 6 (FB6), dermal papilla (DP), immune cell 1 (Immu1), immune cell 2 (Immu2), immune cell 3 (Immu3), endothelial cell (EC), proliferating progenitors (PR), and proliferative progenitor cells (PR). We used CellChat v.1.4.0 to predict the number and weights/strength of intercellular communications from scRNA-seq data. CellCall v. 0.0.0.9000 was utilized to identify the ligand receptor interactions between HM and DP cell clusters. Kyoto Encyclopedia of Genes and Genomes (KEGG) enrichment analyses were performed using an online platform (https://www.bioinformatics.com.cn).

### Low-temperature treatment

The low-temperature treatment was carried out using a handheld device with a thermoelectric cooling surface of approximately 3 cm in diameter. The device can be set to temperatures ranging from 20 ^o^C to 0 ^o^C. Once the temperature is set and stabilized, the thermoelectric cooling surface was held in direct contact with mouse dorsal skin for 5 min. Briefly, the dorsal hair of 3-month-old CD1 mice was removed using the rosin dewaxing method 8. One day into the anagen phase, the cryogenic apparatus was then used to perform localized cutaneous treatment on the backs of the mice at temperatures of 15 ^o^C, 10 ^o^C, 5 ^o^C, and 0 ^o^C. One week later, the back skin of the mice was excised, and sections were stained to observe the expression of relevant factors, and the hair growth of the mice was also recorded.

### Statistical analysis

All data are expressed as the mean ± standard deviation (SD), and differences between samples are analyzed using unpaired two-tailed Student's t tests, multiple t tests with Holm-Šídák method, one-way or two-way analysis of variance (ANOVA) followed by Dunnett's or Šídák* post hoc* tests. P values < 0.05 were considered statistically significant. The exact number of times each experiment was performed is indicated in the figure legends. ImageJ is used for quantifying protein bands and measuring average fluorescence intensity in greyscale. The individual data points are also displayed on the graphs.

## Supplementary Material

Supplementary figures and tables.

## Abbreviations

BSA: Bovine serum albumin
Bulk RNA-seq: RNA sequence of bulk samples
Collagen XVII: Collagen Type XVII Alpha 1 Chain
Col 3a1: Collagen type III alpha 1 chain
CXCL1: C-X-C Motif Chemokine 1
DP: Dermal papilla
DP-ECM: Dermal papilla extracellular matrix
ECM: Extracellular matrix
EMI: Epithelial-mesenchymal interaction
FB: Fibroblast
HM: Hair matrix
HFSCs: Hair follicle stem cells
H&E: Hematoxylin and eosin staining
Igfbp2: Insulin-like growth factor binding proteins 2
KD: Knockdown
LAMA: Laminin
NCBI: National Center for Biotechnology Information
