# Supplementary material for: THSD4 promotes hair growth by facilitating dermal papilla and hair matrix interactions
Source: Theranostics. 2025 Feb 25;15(8):3571–88. doi: 10.7150/thno.103221 (PMC11905124; doi:10.7150/thno.103221)
Supplement: Supplementary file 1 — Supplementary figures and tables. [file thnov15p3571s1.pdf]

THSD4 Promotes Hair Growth by Facilitating Dermal Papilla and Hair Matrix Interactions

Running title: DP-HM crosstalk induces hair growth

Miaomiao Wang[1,2,#], Mengyue Wang[1,#], Jingwei Jiang[1,#], Ke Li[3], Huan Liang[3], Nian'ou Wang[3], Yi Zou[1,4], Dehuan Wang[1], Siyi Zhou[1], Yuchun Tang[1], Wang Wu[1], Weiming Qiu[4], Xinxin Li[5], Xusheng Wang[5], Qiaoli Xie[1], Xiao Xiang[1,*], Wei Zhou[2,*], Li Yang[1,*], Cheng-Ming Chuong[6], Mingxing Lei[1,*]

1. Key Laboratory of Biorheological Science and Technology of Ministry of Education &111 Project Laboratory of Biomechanics and Tissue Repair, College of Bioengineering, Chongqing University, Chongqing 400044, China.

2. Radiation Oncology Center, Chongqing University Cancer Hospital, Chongqing, China.

3. Shenzhen Accompany Technology Cooperation, ltd, Shenzhen 518000, China.

4. Department of Burns and plastic surgery, Wuhan General Hospital of Chinese People's Liberation Army, Wuhan 430000, China.

5. Department of Pathology, Keck School of Medicine, University of Southern California, Los Angeles, CA 90033, USA.

#These authors contributed equally to this work.

Author e-mail Addresses: mingxing@cqu.edu.cn (M.L.); cmchuong@med.usc.edu (C.C.); yanglibme@cqu.edu.cn (L.Y.); 1052308491@qq.com (W.Z.); xiaoxiang_bioeng@cqu.edu.cn (X.X.); qiaolixie@cqu.edu.cn (Q.X.); qweimin87@163.com (W.Q.); wwang@cqu.edu.cn (W.W.);

2419856140@qq.com (Y.T.); 871586143@qq.com (S.Z.); 1536207341@qq.com (D.W.); 838601090@qq.com (Y.Z.); n@accompany.tech (N.W.); lianghuan@accompany.tech (H.L.); like@accompany.tech (K.L.); 1464413215@qq.com (J.J.); 1552766731@qq.com (M.W.); 3398649224@qq.com (M.W.).

Address correspondence to:

Xiao Xiang

Key Laboratory of Biorheological Science and Technology of Ministry of Education &111 Project Laboratory of Biomechanics and Tissue Repair, College of Bioengineering, Chongqing University, Chongqing 400044, China

e-mail: xiaoxiang_bioeng@cqu.edu.cn

Wei Zhou

Radiation Oncology Center, Chongqing University Cancer Hospital, Chongqing, China

e-mail: 1052308491@qq.com

Li Yang

Key Laboratory of Biorheological Science and Technology of Ministry of Education &111 Project Laboratory of Biomechanics and Tissue Repair, College of Bioengineering, Chongqing University, Chongqing 400044, China

e-mail: yanglibme@cqu.edu.cn

Mingxing Lei:

Key Laboratory of Biorheological Science and Technology of Ministry of Education &111 Project Laboratory of Biomechanics and Tissue Repair, College of Bioengineering, Chongqing University, Chongqing 400044, China

e-mail: mingxing@cqu.edu.cn

Supplementary figures

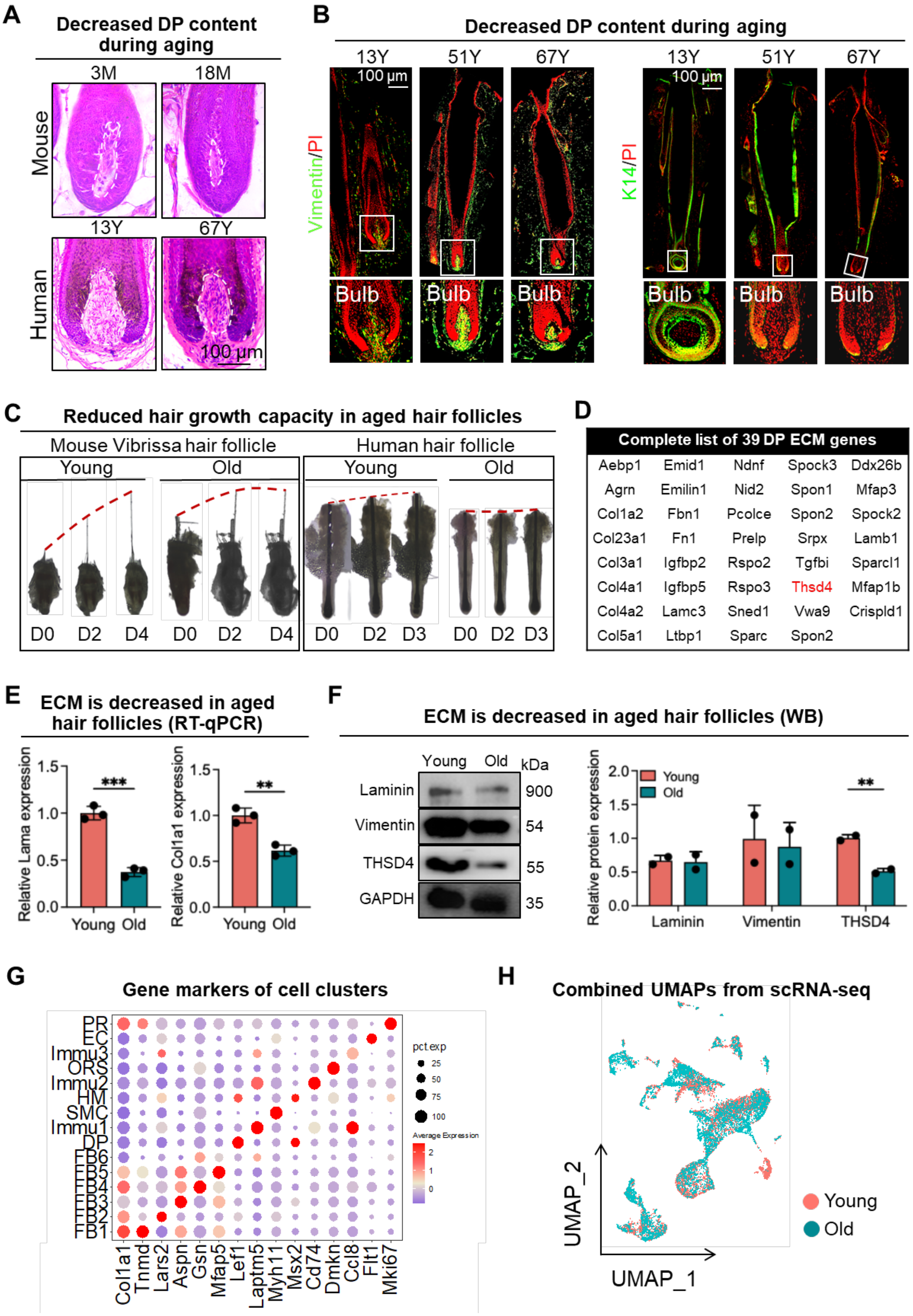

A
Decreased DP content during aging
3M
18M
Mouse
13Y
67Y
Human
100 μm
B
Decreased DP content during aging
13Y
51Y
67Y
100 μm
Vimentin/PI
K14/PI
Bulb
C
Reduced hair growth capacity in aged hair follicles
Mouse Vibrissa hair follicle
Human hair follicle
Young
Old
D0 D2 D4
D0 D2 D3
D
Complete list of 39 DP ECM genes
Aebp1 Emid1 Ndnf Spock3 Ddx26b
Agrn Emilin1 Nid2 Spon1 Mfap3
Col1a2 Fbn1 Pcolce Spon2 Spock2
Col23a1 Fn1 Prelp Srpx Lamb1
Col3a1 Igfbp2 Rspo2 Tgfbi Sparcl1
Col4a1 Igfbp5 Rspo3 Thsd4 Mfap1b
Col4a2 Lamc3 Sned1 Vwa9 Crispld1
Col5a1 Ltbp1 Sparc Spon2
E
ECM is decreased in aged hair follicles (RT-qPCR)
Relative Lama expression
Relative Col1a1 expression
***
**
Young Old
F
ECM is decreased in aged hair follicles (WB)
Young Old kDa
Laminin 900
Vimentin 54
THSD4 55
GAPDH 35
Relative protein expression
Young
Old
**
Laminin
Vimentin
THSD4
G
Gene markers of cell clusters
PR
EC
Immu3
ORS
Immu2
HM
SMC
Immu1
DP
FB6
FB5
FB4
FB3
FB2
FB1
Col1a1 Tnmd Lars2 Aspn Gsn Mfap5 Lef1 Laptm5 Myh11 Msx2 Cd74 Dmkn Ccl8 Flt1 Mki67
pct.exp
25
50
75
100
Average Expression
H
Combined UMAPs from scRNA-seq
UMAP_1
UMAP_2
Young
Old

Figure S1. hair follicle DP content decreases during aging and bulk RNA-sequencing analysis.

A. H&E staining of hair follicles show reduced DP region and content after aging.

B. Immunolabeling of Vimentin and K14 shows overall morphology of human scalp hair follicles during aging. Scale bar = 100 μm.

C. Representative images of ex vivo hair follicles show decreased hair shaft growth upon aging.

D. Table shows entire list of the 39 differentially regulated genes associated with DP ECM. Thsd4 is highlighted in red.

E. qRT-PCR analysis of Lama and Col1a1 expression in the skin of young and old, young and old mice. N=3, ** $p<0.01$, *** $p<0.001$.

F. WB assay showing protein expression levels. N=3, ** $p<0.01$.

G. Marker genes used for unbiased identification of cell clusters during scRNA-seq analysis.

H. Overlay of UMAP plots from young (red) and old (cyan) mouse hair follicles.

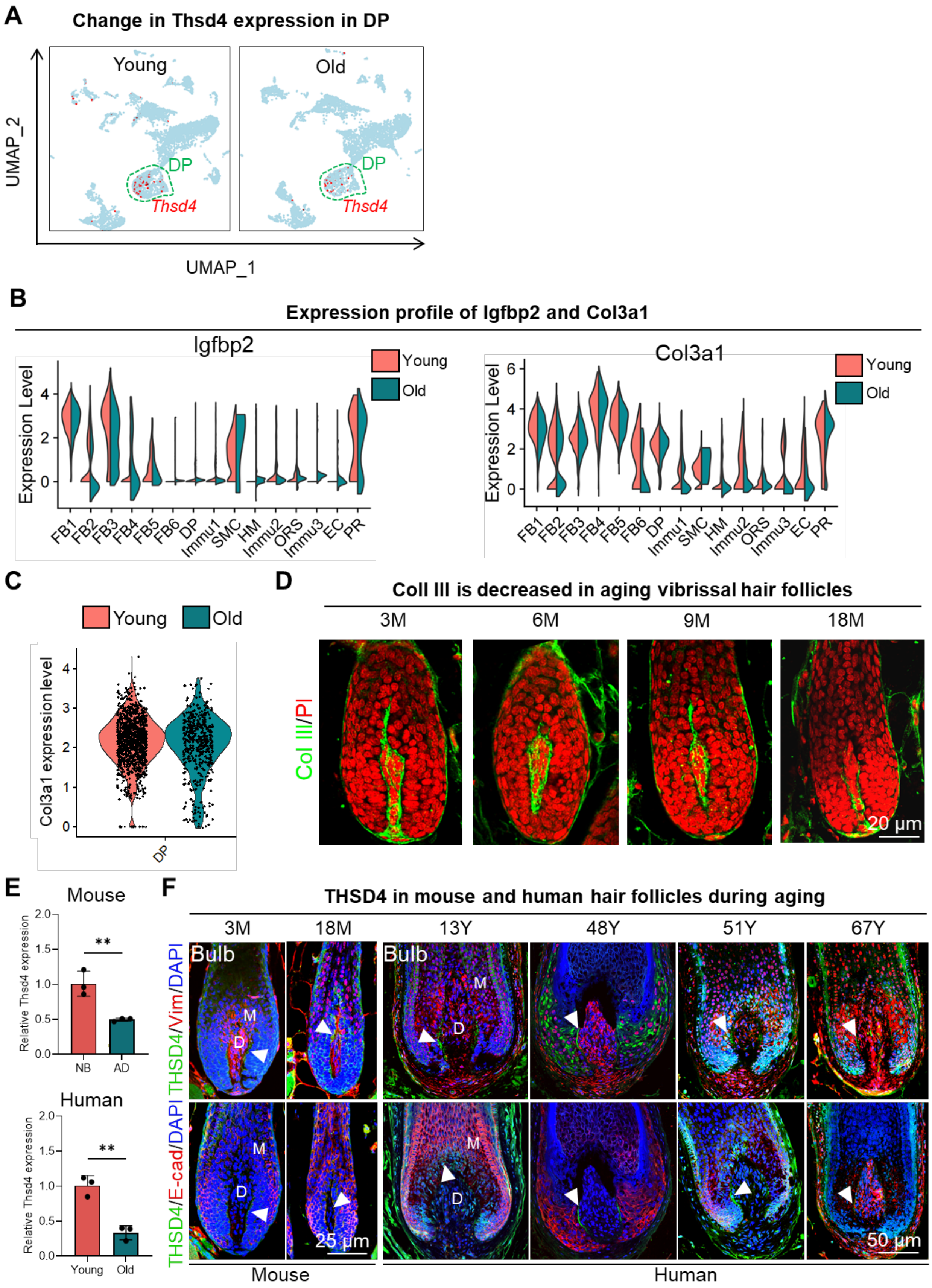
A
Change in Thsd4 expression in DP
Young
Old
DP
Thsd4
UMAP_2
UMAP_1
B
Expression profile of Igfbp2 and Col3a1
Igfbp2
Col3a1
Young
Old
Expression Level
FB1 FB2 FB3 FB4 FB5 FB6 DP Immu1 SMC HM Immu2 ORS Immu3 EC PR
C
Young
Old
Col3a1 expression level
DP
D
Coll III is decreased in aging vibrissal hair follicles
3M
6M
9M
18M
Col III/PI
20 μm
E
Mouse
Relative Thsd4 expression
NB
AD
Human
Young
Old
F
THSD4 in mouse and human hair follicles during aging
3M
18M
13Y
48Y
51Y
67Y
Bulb
M
D
THSD4/Vim/DAPI
THSD4/E-cad/DAPI
25 μm
50 μm
Mouse
Human

Figure S2. Thsd4 is specifically expressed in the DP.

A. UMAP plots show specific detection of Thsd4-positive cells (red) in the DP cell cluster (green circle).

B. Vlnplots show the expression profiles of the top-ranked (log_2 FC > 1.5) DP ECM-related DRGs across various identified cell groups of young and old hair follicles.

C. Bioinformatics analysis shows reduced Col3a1 expression in DP of mouse hair follicles.

D. Quantification shows reduced mouse hair follicle DP content and Col III expression upon aging.

E. qRT-PCR analysis of Thsd4 expression in the skin of young and old, young and old mice. N=3, ** $p<0.01$.

F. Immunostaining shows double staining of Thsd4 with Vimentin and E-cadherin.

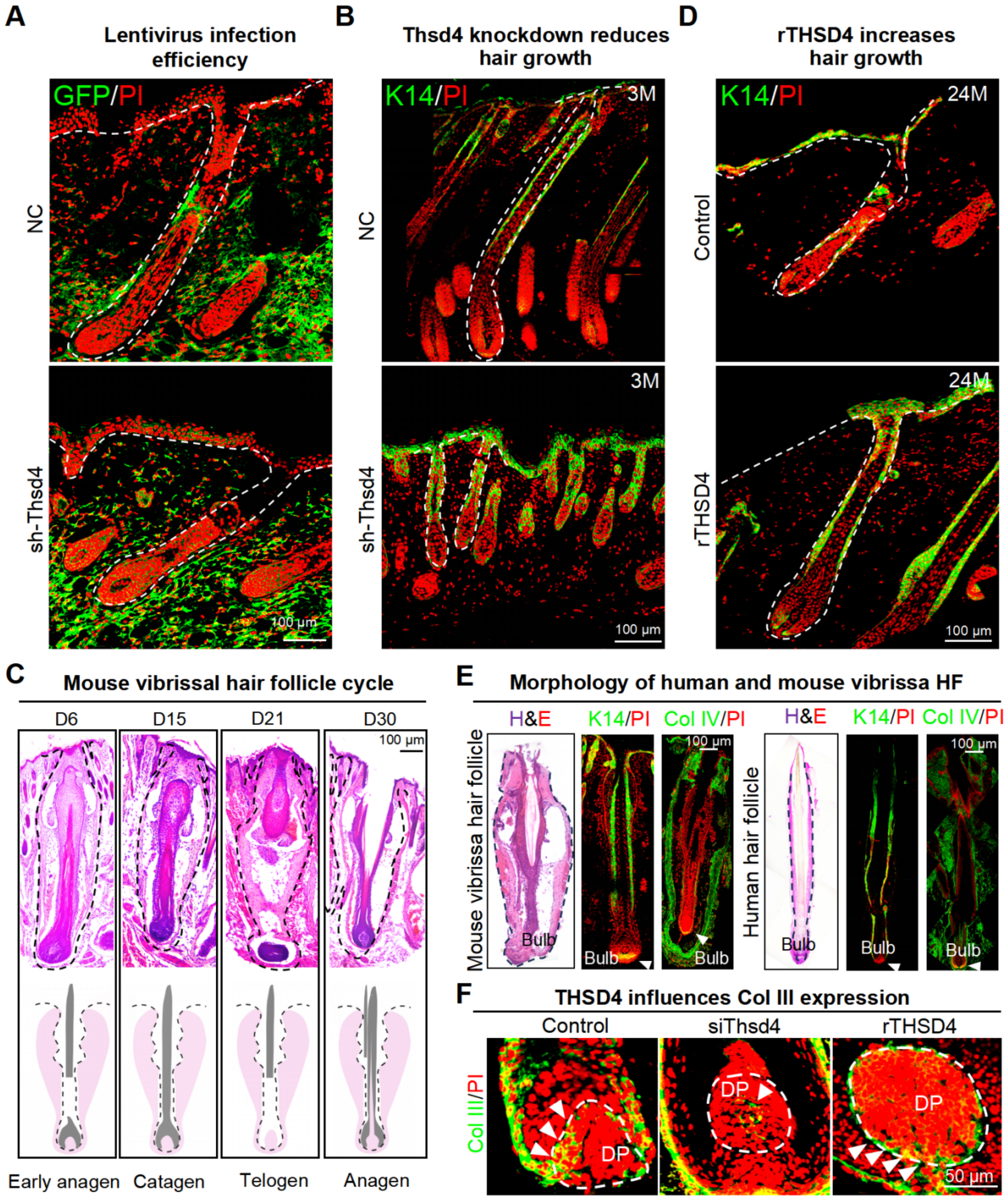
A
Lentivirus infection efficiency
GFP/PI
NC
sh-Thsd4
100 μm
B
Thsd4 knockdown reduces hair growth
K14/PI
3M
NC
sh-Thsd4
100 μm
D
rTHSD4 increases hair growth
K14/PI
24M
Control
rTHSD4
100 μm
C
Mouse vibrissal hair follicle cycle
D6
D15
D21
D30
100 μm
Early anagen
Catagen
Telogen
Anagen
E
Morphology of human and mouse vibrissa HF
H&E
K14/PI
Col IV/PI
Mouse vibrissa hair follicle
Human hair follicle
Bulb
100 μm
F
THSD4 influences Col III expression
Control
siThsd4
rTHSD4
Col III/PI
DP
50 μm

Figure S3. Morphological characterization of hair follicles.

A. Immunofluorescence images show successful delivery of Thsd4-targeting shRNA or its non-targeting control (NC), indicated by GFP fluorescence, via adenoviral infection of murine dorsal skin. Scale bar = 100 μm.

B. Immunofluorescence images show reduction in hair follicle growth after Thsd4 knockdown in young mouse. Scale bar = 100 μm.

C. H&E staining and schematic depiction show morphological change of mouse vibrissal hair follicle during different growth phases. Scale bar = 100 μm.

D. Immunofluorescence images show increase in hair follicle growth after treatment with recombinant THSD4 in aged mouse. Scale bar = 100 μm.

E. H&E and immunofluorescence images show morphologies of extracted mouse vibrissal and human scalp hair follicles. The overall morphologies were preserved after extraction. Scale bar = 100 μm.

F. Immunostaining of Col III in mouse vibrissal hair follicles after modulation of Thsd4 levels. Arrowheads point at the Col III-positive regions in DP. Scale bar = 50 μm.

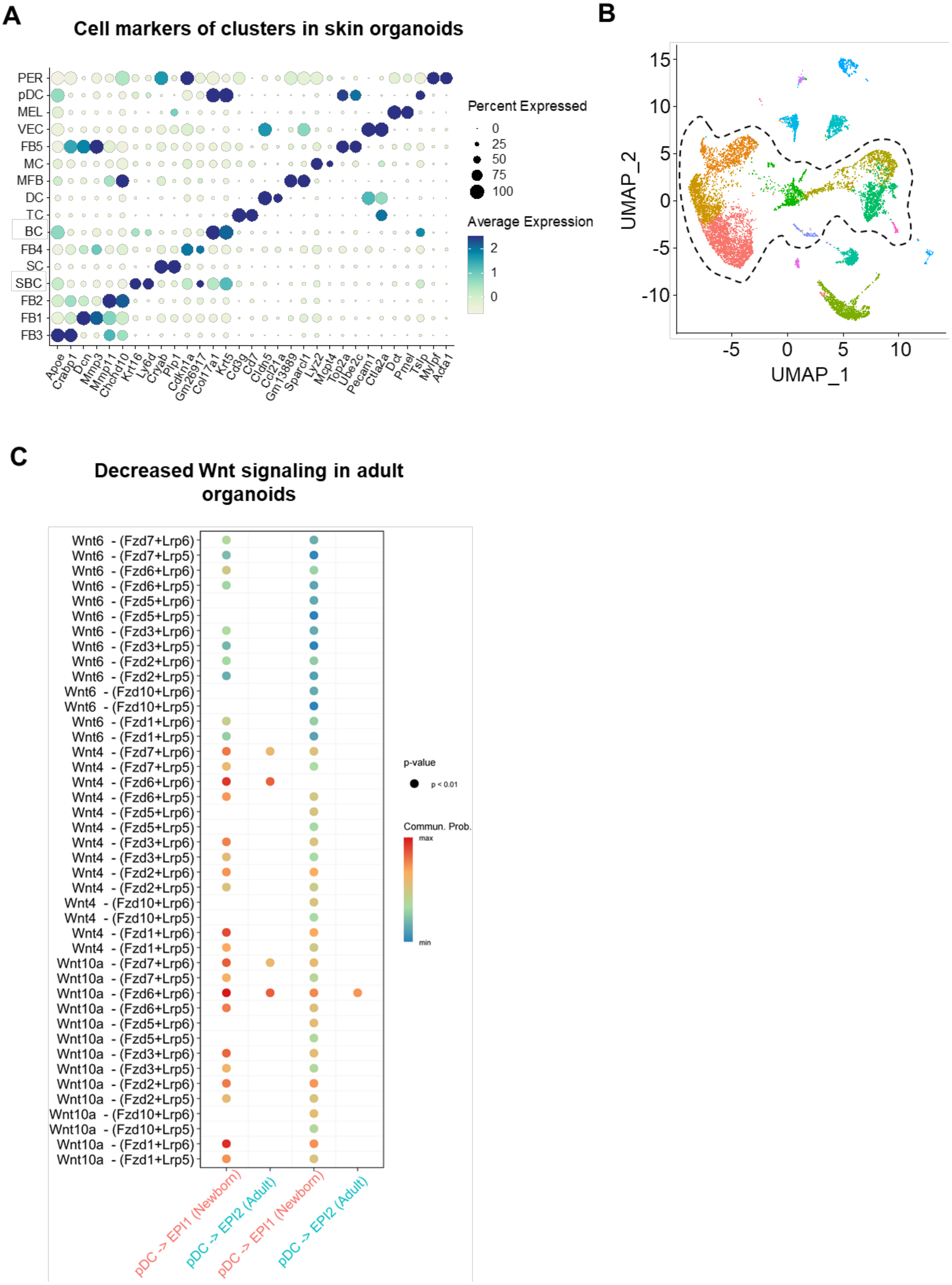

A
Cell markers of clusters in skin organoids
PER
pDC
MEL
VEC
FB5
MC
MFB
DC
TC
BC
FB4
SC
SBC
FB2
FB1
FB3
Apoe
Crabp1
Dcn
Mmp3
Mmp11
Chchd10
Krt16
Ly6d
Cryab
Plp1
Cdkn1a
Gm26917
Col17a1
Krt5
Cd3g
Ccl7
Cldn5
Ccl21a
Gm13889
Sparcl1
Lyz2
Mcpt4
Top2a
Ube2c
Pecam1
Ctla2a
Dct
Pmel
Tslp
Mylpf
Acta1
Percent Expressed
0
25
50
75
100
Average Expression
2
1
0
B
UMAP_2
UMAP_1
15
10
5
0
-5
-10
-5
0
5
10
C
Decreased Wnt signaling in adult organoids
Wnt6 - (Fzd7+Lrp6)
Wnt6 - (Fzd7+Lrp5)
Wnt6 - (Fzd6+Lrp6)
Wnt6 - (Fzd6+Lrp5)
Wnt6 - (Fzd5+Lrp6)
Wnt6 - (Fzd5+Lrp5)
Wnt6 - (Fzd3+Lrp6)
Wnt6 - (Fzd3+Lrp5)
Wnt6 - (Fzd2+Lrp6)
Wnt6 - (Fzd2+Lrp5)
Wnt6 - (Fzd10+Lrp6)
Wnt6 - (Fzd10+Lrp5)
Wnt6 - (Fzd1+Lrp6)
Wnt6 - (Fzd1+Lrp5)
Wnt4 - (Fzd7+Lrp6)
Wnt4 - (Fzd7+Lrp5)
Wnt4 - (Fzd6+Lrp6)
Wnt4 - (Fzd6+Lrp5)
Wnt4 - (Fzd5+Lrp6)
Wnt4 - (Fzd5+Lrp5)
Wnt4 - (Fzd3+Lrp6)
Wnt4 - (Fzd3+Lrp5)
Wnt4 - (Fzd2+Lrp6)
Wnt4 - (Fzd2+Lrp5)
Wnt4 - (Fzd10+Lrp6)
Wnt4 - (Fzd10+Lrp5)
Wnt4 - (Fzd1+Lrp6)
Wnt4 - (Fzd1+Lrp5)
Wnt10a - (Fzd7+Lrp6)
Wnt10a - (Fzd7+Lrp5)
Wnt10a - (Fzd6+Lrp6)
Wnt10a - (Fzd6+Lrp5)
Wnt10a - (Fzd5+Lrp6)
Wnt10a - (Fzd5+Lrp5)
Wnt10a - (Fzd3+Lrp6)
Wnt10a - (Fzd3+Lrp5)
Wnt10a - (Fzd2+Lrp6)
Wnt10a - (Fzd2+Lrp5)
Wnt10a - (Fzd10+Lrp6)
Wnt10a - (Fzd10+Lrp5)
Wnt10a - (Fzd1+Lrp6)
Wnt10a - (Fzd1+Lrp5)
p-value
p < 0.01
Commun. Prob.
max
min
pDC -> EPI1 (Newborn)
pDC -> EPI2 (Adult)
pDC -> EPI1 (Newborn)
pDC -> EPI2 (Adult)

Figure S4. ScRNA-sequencing analysis of epithelial and dermal cells.

A. Marker genes used for unbiased identification of cell clusters, particularly the epithelial clusters (FB1-4) and dermal cell clusters (SBC and BC), during scRNA-seq analysis.

B. UMAP plot from mouse skin organoids on D4 showing all identified cell clusters.

C. CellChat analyses of signaling pathways of pDC interaction with dermis and epidermis.

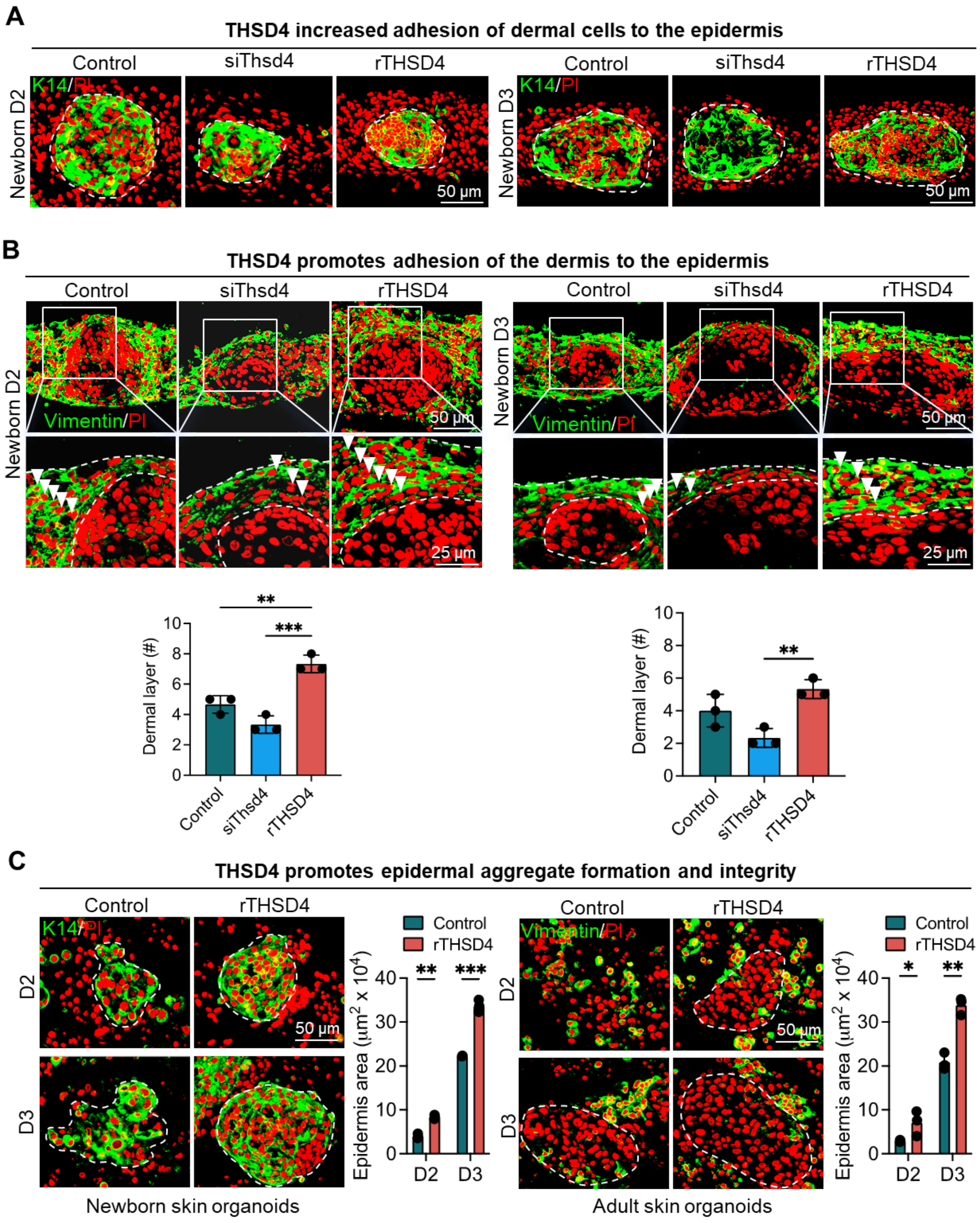

Figure S5. THSD4 enhances dermal adhesion to epidermis.

A. Immunostaining of K14 in newborn mouse skin organoids show regulation of dermal cell adhesion by THSD4. Scale bar = 50 μm.

B. Immunostaining of Vimentin in newborn mouse skin organoids and quantification show regulation of dermal cell adhesion by Thsd4. N=3, ** $p<0.01$, *** $p<0.001$. Upper panel: scale bar = 50 μm; lower panel: scale bar = 25 μm.

C.Immunofluorescence images of adult mouse skin organoids and quantification show increased dermal aggregation after Thsd4 treatment. N=3, * $p<0.05$, ** $p<0.01$, *** $p<0.001$. Scale bar = 50 μm.

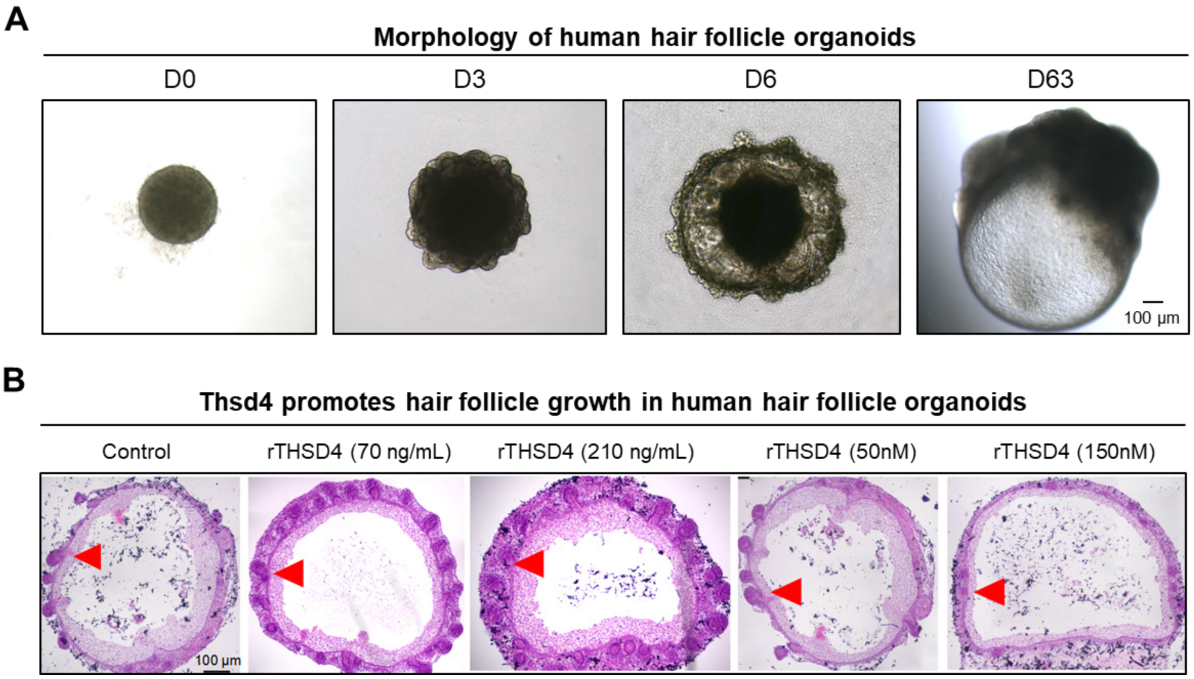

Figure S6. Thsd4 promotes hair follicle growth in human hair follicle organoids.

A. Morphology of human hair follicle organoids.

B. H&E staining showing the morphology of hair follicle growth in organoids of human origin.

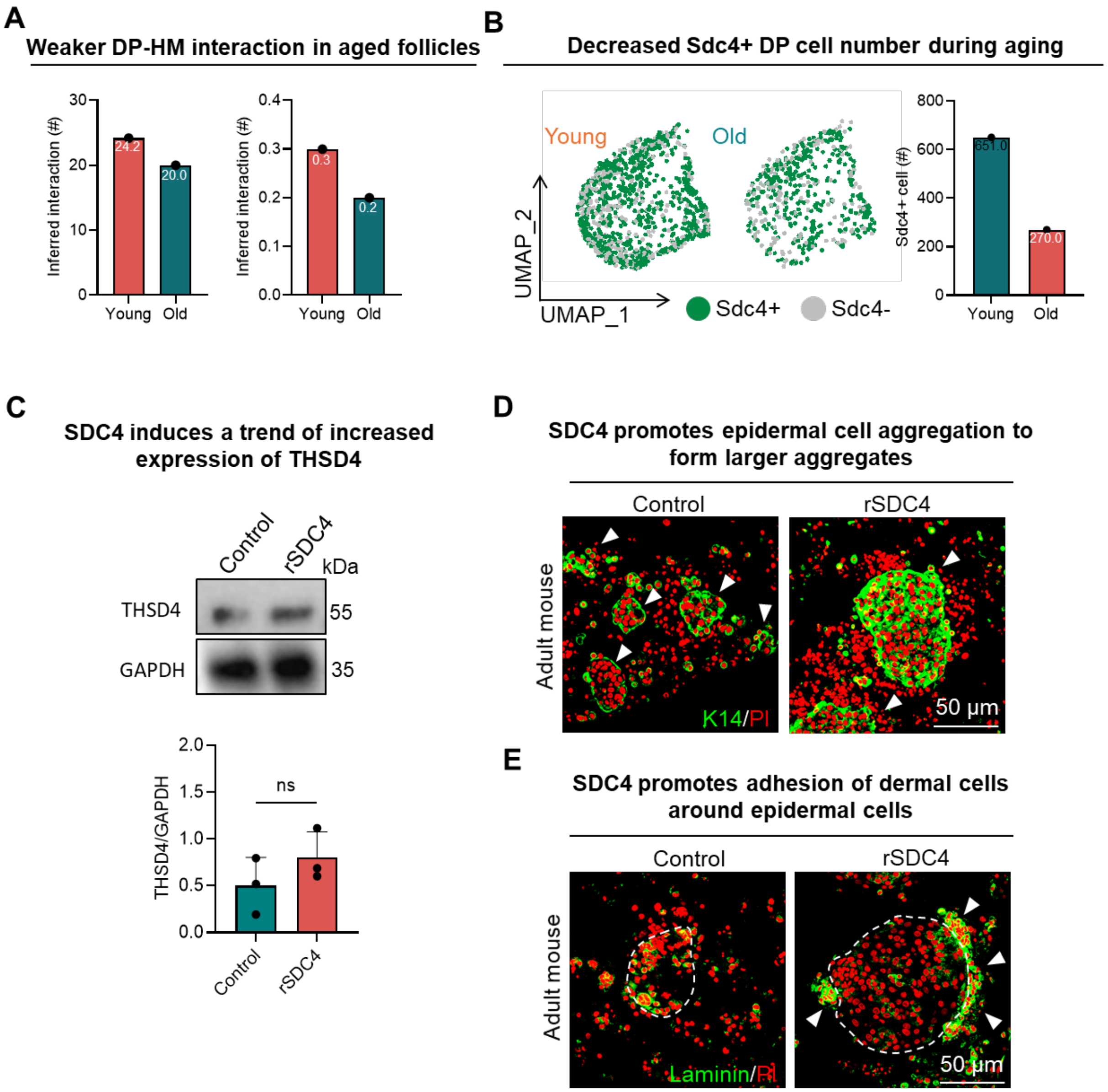

Figure S7. Characterization of SDC4-THSD4 signaling stimulated by HM.

A. Quantification of hair matrix (HM) / DP signal as in number of interaction and signal strength.

B. Feature plot and quantification show reduced SDC4+ cell numbers upon aging.

C. Western Blot experiments demonstrate the protein expression levels of the Control group with rSDC4 and its quantification, N=3, ns = no significance.

D. Immunostaining of K14 in adult mouse skin organoids show increased dermal aggregates after treatment with recombinant SDC4. Scale bar = 50 μm.

E. Immunostaining of Laminin in adult mouse skin organoids shows increased dermal adhesion following SDC4 treatment. Scale bar = 50 µm.

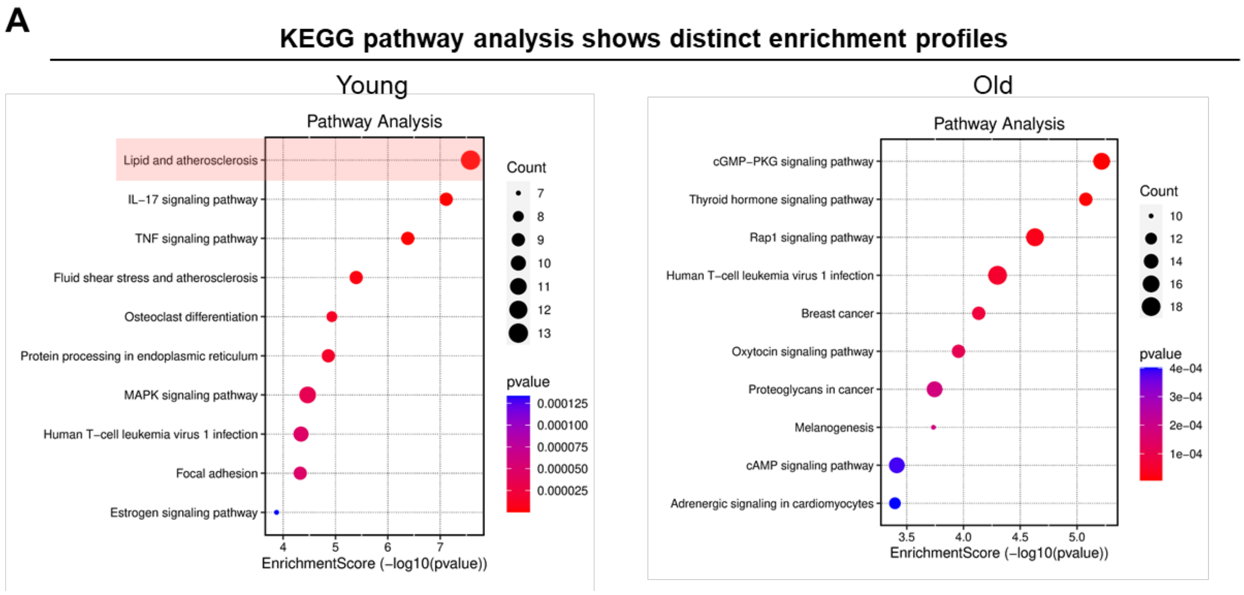

Figure S8. KEGG pathway analyses of bulk RNA-seq from hair follicles of young and old mice.

A. KEGG pathway analyses show distinct enrichment profiles between young and old murine hair follicles.

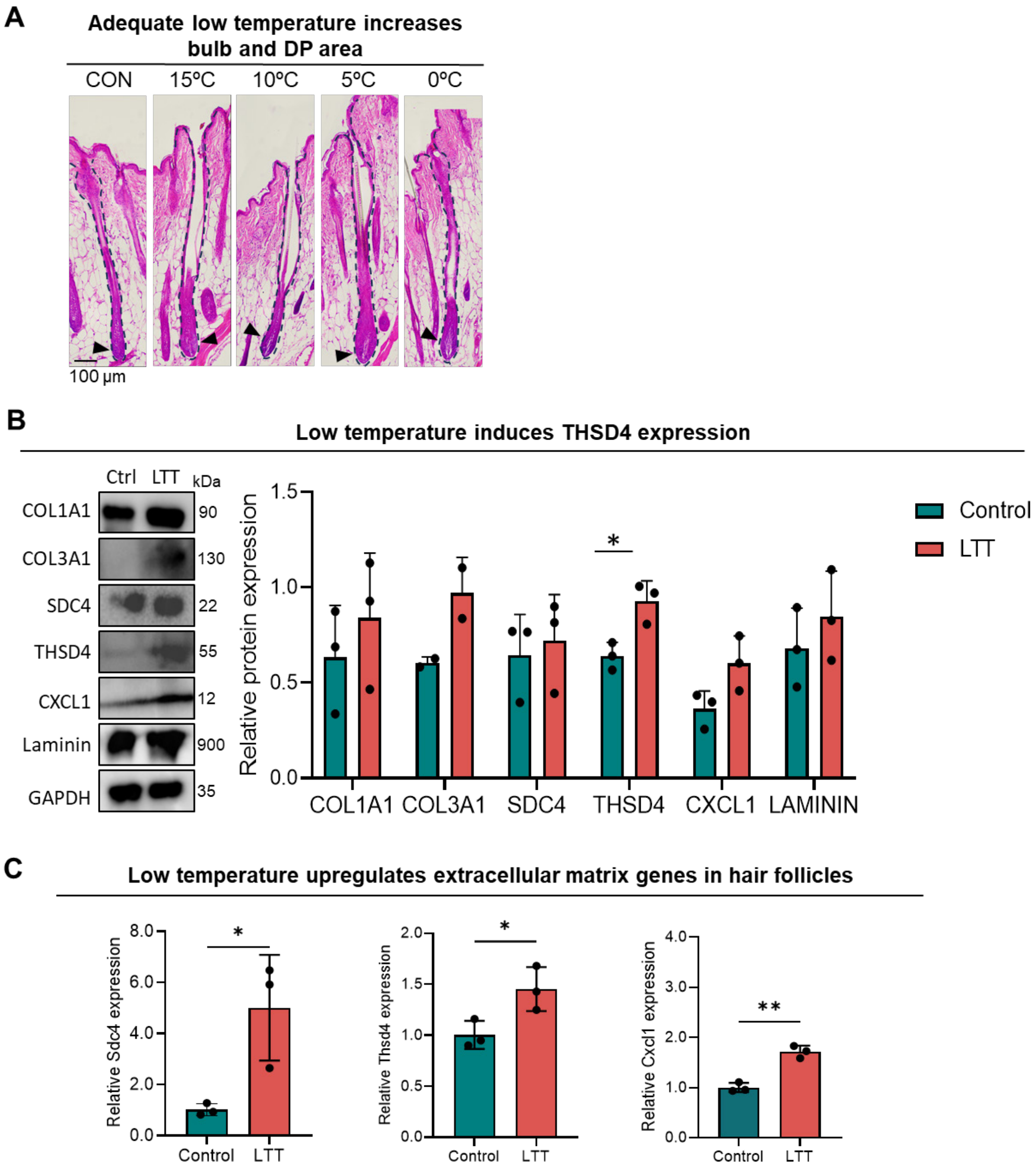

Figure S9. Low-temperature treatment increases matrix cell proliferation.

A. H&E staining of mouse hair follicles after 5-minute low-temperature treatments (LTT). Arrowheads indicate hair bulbs. Scale bar = 100 µm.

B. Statistical WB protein banding data to compare relative protein expression. N=3, * $p<0.05$.

C. Statistical q-RT PCR data to compare relative gene expression. N=3, ** $p<0.01$, * $p<0.05$.

Supplementary tables

Supplementary Table 1. List of primer sequences for Real-time qPCR.

**qRT-PCR Primers**		
**Gene Name**	**Forward (5' to 3')**	**Reverse (5' to 3')**
Gapdh	CTGGGCTACACTGAGCACC	AAGTGGTCGTTGAGGGCAATG
Thsd4	GCTGCCCTTGACTCACGAC	AAGTTGTCTGGTCGGTGGG
Cxcl1	CTGGGATTCACCTCAAGAACATC	CAGGGTCAAGGCAAGCCTC
Col1a1	TTCCCGGTGAATTCGGTCTC	ACCTCGGATTCCAATAGGACCAG
Col3a1	TTTGGCACAGCAGTCCAATGTA	GACAGATCCCGAGTCGCAGA
Sdc4	ATGACTTTGAGCTCTCGGGTTCT	TGCATTCTCAGGGATGTGGTTAT
Lama	TCAATCAGGACCGCTTCATC	CGTGAGCAATCTTCTCACTG

Supplementary Table 2. List of antibodies used in this study.

Antibody	Isotype	Company	Cat #
Col I	Rabbit	ZenBio	252577
Col III	Rabbit	Proteintech	22734-1-AP
CXCL1	Rabbit	Proteintech	12335-1-AP
GFP	Rabbit	Zenbio	380956
E-cadherin	mouse	Beyotime	AF0138
KRT14	Mouse	Boster	A01432
LAMA	Mouse	Abcam	Ab11575
LEF1	Rabbit	Beyotime	380956
PCNA	Rabbit	Beyotime	E-AB-22001
P63	Rabbit	Gentex	GTX102425
SDC4	Rabbit	Proteintech	11820-1-AP
THSD4	Rabbit	Proteintech	20619-1-AP
Tublin	Mouse	CST	5568T
Vimentin	Mouse	Beyotime	AF0318
Rabbit anti-Goat IgG	Rabbit	Zsbio	ZF-0314
Mouse anti-Goat IgG	Mouse	Beyotime	C1002
